# A Bayesian non-inferiority approach using experts’ margin elicitation – application to the monitoring of safety events

**DOI:** 10.1186/s12874-019-0826-5

**Published:** 2019-09-18

**Authors:** Camille Aupiais, Corinne Alberti, Thomas Schmitz, Olivier Baud, Moreno Ursino, Sarah Zohar

**Affiliations:** 1 Inserm, U1138, Equipe 22, Centre de Recherche des Cordeliers, Sorbonne University, University Paris Descartes, 15 rue de l’École de médecine, Paris, 75006 France; 2 University Paris Diderot, Site Villemin, 10 avenue de Verdun, Paris, 75010 France; 3 Inserm, U1123, ECEVE, 10 avenue de Verdun, Paris, 75010 France; 4 Unité d’épidémiologie clinique, CIC-EC 1426, Assistance Publique-Hôpitaux de Paris, Hôpital Robert Debré, 48 boulevard Sérurier, Paris, 75019 France; 5 Service de Gynécologie Obstétrique, Assistance Publique-Hôpitaux de Paris, Hôpital Robert Debré, 48 boulevard Sérurier, Paris, 75019 France; 6 Inserm, U1153, Epidemiology and Biostatistics Sorbonne Paris Cité Research Center, Obstetrical, Perinatal and Pediatric Epidemiology Team, 53 avenue de l’observatoire, Paris, 75014 France; 7 Service de néonatalogie, Assistance Publique-Hôpitaux de Paris, Hôpital Robert Debré, 48 boulevard Sérurier, Paris, 75019 France; 8 Inserm, U1141, Hôpital Robert Debré, 48 boulevard Sérurier, Paris, 75019 France; 9 (Present address) Service de néonatalogie, Hôpitaux universitaires de Genève, 32 boulevard de la Cluse, Genève, 1205 Suisse; 10 F-CRIN PARTNERS Platform (AP-HP), 10 avenue de Verdun, Paris, 75010 France

**Keywords:** Clinical trial, Non-inferiority, Bayesian inference, Mixture model, Children, Elicitation

## Abstract

**Background:**

When conducing Phase-III trial, regulatory agencies and investigators might want to get reliable information about rare but serious safety outcomes during the trial. Bayesian non-inferiority approaches have been developed, but commonly utilize historical placebo-controlled data to define the margin, depend on a single final analysis, and no recommendation is provided to define the prespecified decision threshold. In this study, we propose a non-inferiority Bayesian approach for sequential monitoring of rare dichotomous safety events incorporating experts’ opinions on margins.

**Methods:**

A Bayesian decision criterion was constructed to monitor four safety events during a non-inferiority trial conducted on pregnant women at risk for premature delivery. Based on experts’ elicitation, margins were built using mixtures of beta distributions that preserve experts’ variability. Non-informative and informative prior distributions and several decision thresholds were evaluated through an extensive sensitivity analysis. The parameters were selected in order to maintain two rates of misclassifications under prespecified rates, that is, trials that wrongly concluded an unacceptable excess in the experimental arm, or otherwise.

**Results:**

The opinions of 44 experts were elicited about each event non-inferiority margins and its relative severity. In the illustrative trial, the maximal misclassification rates were adapted to events’ severity. Using those maximal rates, several priors gave good results and one of them was retained for all events. Each event was associated with a specific decision threshold choice, allowing for the consideration of some differences in their prevalence, margins and severity. Our decision rule has been applied to a simulated dataset.

**Conclusions:**

In settings where evidence is lacking and where some rare but serious safety events have to be monitored during non-inferiority trials, we propose a methodology that avoids an arbitrary margin choice and helps in the decision making at each interim analysis. This decision rule is parametrized to consider the rarity and the relative severity of the events and requires a strong collaboration between physicians and the trial statisticians for the benefit of all. This Bayesian approach could be applied as a complement to the frequentist analysis, so both Data Safety Monitoring Boards and investigators can benefit from such an approach.

**Electronic supplementary material:**

The online version of this article (10.1186/s12874-019-0826-5) contains supplementary material, which is available to authorized users.

## Background

Non-inferiority (NI) randomized clinical trials aim to demonstrate whether an experimental treatment is not inferior, below a certain pre-specified margin, to the control treatment [1]. This margin should be formulated according to earlier knowledge and clinical relevance [1, 2]. It has been shown, for instance in paediatrics, that the choice is not well-documented in 63% of studies [3]. However, when there is no reliable placebo-controlled historical data, and when conducting such a trial is not ethical due to changes in practices, margins based solely on clinical judgement could be acceptable, if constructed with rigorous methods, such as systematic analysis of several independent experts’ opinions.

When conducing a trial, the analysis of some secondary outcomes, in addition to the primary endpoint, might be challenging, as the sample size was not specifically tuned for that. This issue is of particular importance for safety events, and is even more true when considering rare but critical safety outcomes, which might not occur or only a few can be observed. Consequently, these individual trials are usually under-powered to detect safety differences and to ensure reliable conclusions. Some efficient methods have been proposed for the detection of rare events that are using meta-analysis tools in order to improve overall power. Nevertheless, many methods of meta-analysis are based on large sample approximations, and may be unsuitable when events are rare [4]. Moreover, regulatory agencies and investigators may not wish to wait for post-marketing studies to draw conclusions about rare but serious outcomes of a new intervention. Furthermore, they might want to get reliable safety information before the end of a trial.

When considering NI trials, investigators would like to monitor whether the difference in safety outcomes between arms is clinically relevant. In this case, similar reasoning as for the efficacy primary outcome can be applied, using specific NI margins. If we consider settings where events are rare, a Bayesian approach may seem appropriate to construct sequential stopping rules. Several authors have proposed Bayesian designs for NI trials [5, 6]. Gamalo et al. have proposed a Bayesian NI approach for binary endpoints in which an active-control’s treatment effect is estimated using historical data under a fixed margin assumption [7]. However, this Bayesian decision criterion utilizes historical placebo-controlled data, it depends on a single final analysis, and no recommendation is provided to define the prespecified decision threshold.

We propose a Bayesian NI sequential design to monitor several safety dichotomous events where margins are based on clinical relevance obtained from several experts.

### Motivation

The ongoing BETADOSE study (NCT02897076) aims to demonstrate that a 50% reduced betamethasone dose regimen is not inferior to a full-dose in preventing neonatal severe respiratory distress syndrome [8]. Several studies have proven the benefice of antenatal corticosteroids, such as betamethasone, so it is used worldwide in pregnant women at risk [9–13]. However, concerns persist regarding long-term adverse events of antenatal corticosteroids, mainly dose-related [14–16].

The trial plans to include 1571 women per arm in 37 French centres. A sequential data analysis has been planned after every 300 newborns reach the primary outcome.

As a safety secondary objective, the protocol plans to monitor, at each interim analysis, the absence of an excess of four other neonatal complications, i.e., neonatal death, severe intraventricular haemorrhage (IVH), necrotising enterocolitis and retinopathy, in two gestational age subgroups of neonates (<28 weeks, 28–32 weeks).

Because only 33% of the randomized women are expected to deliver before 32 weeks, and due to the low frequency of some complications in preterm children, the trial planning had to cope with an expected low number of some secondary events (based on the EPIPAGE-2 cohort study - Additional file 1) [17]. As a consequence, standard frequentist analysis of those outcomes, consisting of tests repeated at each interim analysis, might be powerless.

The Bayesian approach proposed in the manuscript will be applied to this trial (complementary to the frequentist analysis) so the Data Safety Monitoring Board and the investigators can evaluate the difference in terms of the result’s interpretation and the benefit of such an approach.

## Methods

**Fig. 1:**
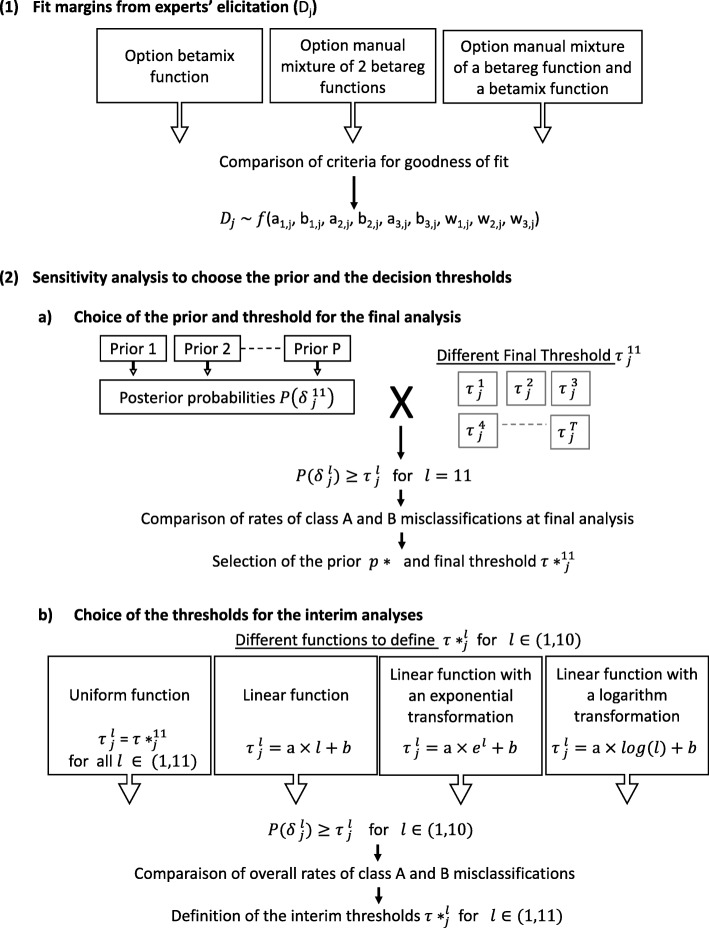
General framework describing the two steps of the decision rule building. This figure summarizes the general framework, divided in two steps: **1** Fit margins from experts’ elicitation (Dj); **2** Sensitivity analysis to choose the prior and the decision thresholds

Let *i*=0,1 be the arm-index (1 for the half-dose, 0 for the full-dose) and *j*=1,2,3,4 the event-index. For the sake of clarity, we show the methodology and results for only one subgroup of neonates (<28 weeks), as it can be repeated in the other subgroup. We used a Bayesian Non-Inferiority approach, detailed in the next subsection. If *π*_*i,j*_ denotes the event rate in the *i*^*th*^ arm, and *D*_*j*_∈(0,1) the maximal acceptable difference, the probabilities of interest are *Pr*(*π*_1,*j*_−*π*_0,*j*_≤*D*_*j*_). To consider the difference in event prevalence and relative severities, this approach was done for each event (*j*). In our setting, the quantity *D*_*j*_ is not a fixed value, but rather a distribution fitted from elicited experts’ opinions through a mixture of beta distributions to consider some variability between experts. The setting of prior distributions and decision thresholds are detailed in the following subsections. Then, a practical example is given using a simulated dataset that mimics the trial. A summary of the general framework is presented in Fig. 1.

### Bayesian non-inferiority approach

For each event *j* and arm *i*, let *y*_*i,j*,*n*_ denote the observed binary outcome for the *n*^*th*^ subject, *n*_*i*_ the total number of observations and $Y_{i,j} = \sum _{n=0}^{n_{i}}{y_{i,j,n}} $ the number of events. Following a Bayesian binomial model, we have

1 $$ Y_{i,j} \sim Bin(n_{i}, \theta_{i,j})   $$

where *θ*_*i,j*_∼*Beta*(*α*_*i,j*_,*β*_*i,j*_) are considered as random variables following a beta prior density. In this setting, the posterior distribution of each *θ*_*i,j*_ is given by:

2 $$ \theta_{i,j} \vert Y_{i,j} \sim Beta(\alpha_{i,j} + Y_{i,j}, \beta_{i,j} + n_{i,j} - Y_{i,j})   $$

Indexing by *l* the interim analysis, *l*∈[1,…,*L*], we will calculate for each event at each analysis the posterior probability that the difference of events rates, *θ*_1,*j*_−*θ*_0,*j*_, is higher than the acceptable difference distribution *D*_*j*_:

3 $$\begin{array}{*{20}l} {}P \left(\delta^{l}_{j}\right) & = P \left(\theta_{1,j} - \theta_{0,j} > D_{j} \ \vert \ Y^{l}_{1,j}, Y^{l}_{0,j}\right)  \\ & = \!\int_{0}^{1}\! \left(\!\theta_{1,j} \,-\, \theta_{0,j} \!>\! x \ \!\vert\! \ Y^{l}_{1,j}, Y^{l}_{0,j}, D_{j}=x\! \right)\! \ . \ P(D_{j}=x)\! \ . \, \text{dx}  \end{array} $$

At the *l*^*th*^ interim analysis, the Bayesian decision rule will conclude that there is an unacceptable excess of event *j* in the experimental arm if $P (\delta ^{l}_{j}) \geq \tau ^{l}_{j}$, where $\tau ^{l}_{j}$ is a prespecified decision threshold.

### Fit margins from experts’ elicitation

To evaluate the distribution of *D*_*j*_, the acceptable difference of events rate between arms, we performed a formal elicitation with several experts. A questionnaire was sent to the two main investigators (1 obstetrician and 1 neonatologist) of each centre involved in the trial. They were asked about (i) their own characteristics (age, sex, speciality, etc.), (ii) the maximum prevalence of events they may tolerate in the experimental arm, given the expected prevalence of each event in the control arm, (iii) the weight of each event, that is the relative severity of the outcomes, considering that death has maximum weight equal to 100.

Let $\tilde {f}_{j} $ denote the estimated event rate in the full-dose arm, based on the EPIPAGE-2 study (Additional file 1), and *h*_*j,e*_ the acceptable event rate in the half-dose arm according to the *e*^*th*^ expert, *e*∈[1,…,*E*]. The acceptable difference between arms according to the *e*^*th*^ expert is: $d_{j,e} = h_{j,e} - \tilde {f}_{j} $. For each event, the distribution of the acceptable difference among the *E* experts was modeled using a mixture of beta distributions, with a maximum of 3 distributions. Using the betamix function (betareg package on R software [18, 19]), 3 different estimation methods were adopted (the first mathematically driven and the other two empirically driven). See Table 1 for details as well as Section 1 of the Additional file 7. As results, the distribution of *D*_*j*_ will be denoted as *D*_*j*_∼*f*(*a*_1,*j*_,*b*_1,*j*_,*a*_2,*j*_,*b*_2,*j*_,*a*_3,*j*_,*b*_3,*j*_,*w*_1,*j*_,*w*_2,*j*_,*w*_3,*j*_), where (*a*_1,*j*_,*b*_1,*j*_), (*a*_2,*j*_,*b*_2,*j*_) and (*a*_3,*j*_,*b*_3,*j*_) are parameters for the 3 *beta* distributions, and (*w*_1,*j*_,*w*_2,*j*_,*w*_3,*j*_) the corresponding weights. Parameters will be omitted when mixtures contain less than 3 distributions.

**Table 1:** Three methods of fitting used to model the physicians’ acceptable differences of rates of events

1	*Option*betamix*function*: For each pair (*j,k*), application of the betamix function with 3 as maximal number of components of the finite mixture.
2	*Option manual mixture of 2*betareg*function*: The levels of the observed values of *d*_*j,k*,*e*_ were dichotomized. Then, we fit 2 *Beta* distribution by applying the betareg function (or the equivalent betamixfunction with 1 as the number of components) on each level of dichotomisation. All levels of dichotomisation were compared, from that separating the two left values from the others, to that separating the two right values from the others. The two distributions were then mixed by applying the weights *w*_1,*j*_ and *w*_2,*j*_=1−*w*_1,*j*_ to each distribution. The weights *w*_1,*j*_∈(0,0.05,0.10,0.15,…,0.95,1) were tested. The models obtained with the different levels of dichotomisation and with the different weights were compared using the criteria for goodness of fit described in Section 1 of the Additional file 7. The fit with the lowest criteria was retained for the comparison with the other 2 methods.
3	*Option manual mixture of a*betamix*function and a*betareg*function*: A mixture of betamix function and *manual mixture*: We mixed: (i) a first *Beta* distribution obtained on the left level of dichotomisation (the one obtained with method 2), (ii) a mixture of a second and a third distribution, obtained by applying to the right level of dichotomisation the betamix function with 2 as the number of components. The weights given to those distributions were: (i) for the first distribution the *w*_1,*j*_ was obtained through method 2, (ii) for the second and third distribution, the weights *w*_2,*j*_ and *w*_3,*j*_ were obtained through the ’betamix’ procedure, multiplied by (1−*w*_1,*j*_).

### Sensitivity analysis to select the prior and the decision thresholds

The sensitivity analysis aimed to compare the performances of different priors and thresholds $\tau ^{l}_{j}$ and to select the most appropriate combination. In the reference arm, *θ*_0,*j*_ was imputed from historical data (Table 2) [17]. For the experimental arm, five scenarios were considered, determined by the assumed true values of the response probabilities (*θ*_1,*j*_). Let *s* be the scenario-index (*s*∈[1,…,5]), and *θ*_1,*j,s*_ denote the prevalence in the experimental arm of the *s*^*th*^ scenario. In the first scenario, the prevalence in the experimental and control arms are the same (*θ*_1,*j*,1_=*θ*_0,*j*_). In the second scenario, the prevalence are lower in the experimental than in the control arm (*θ*_1,*j*,2_=2/3×*θ*_0,*j*_). In the third to fifth scenario, the prevalence is higher in the experimental than in the control arm (*θ*_1,*j*,3_=1.5×*θ*_0,*j*_, *θ*_1,*j*,4_=2×*θ*_0,*j*_ and *θ*_1,*j*,5_=3×*θ*_0,*j*_). For each scenario, 1000 trials have been generated, with *n*_*i*_=162 (Additional file 1), and *Y*_*i,j*,*s*_ following the Eq. (1).

**Table 2:** Prevalence of events assumed in each trial, according to the scenario and to the application data set, and weight and maximal rates of misclassifications assigned to each event to build the decision rule

		Event			
		Death	IVH ^[1]^	NEC ^[2]^	Retinopathy
Simulation study					
All scenarios	Prevalence in FD arm	0.39	0.15	0.06	0.04
Scenario A	Prevalence in HD arm	0.39	0.15	0.06	0.04
	Good decision ^[3]^	Acc	Acc	Acc	Acc
Scenario B	Prevalence in HD arm	0.26	0.10	0.04	0.03
	Good decision ^[3]^	Acc	Acc	Acc	Acc
Scenario C	Prevalence in HD arm	0.58	0.23	0.09	0.06
	Good decision ^[3]^	U	U	Acc	Acc
Scenario D	Prevalence in HD arm	0.78	0.30	0.12	0.08
	Good decision ^[3]^	U	U	U	U
Scenario E	Prevalence in HD arm	1.00	0.45	0.18	0.12
	Good decision ^[3]^	U	U	U	U
Weight^[4]^		100	88	70	60
Maximal misclassifications rates					
Class *a* misclassifications ^[5]^		0.10	0.10	0.10	0.10
Class *b* misclassifications ^[6]^		0.10	0.16	0.25	0.30
Data set for application					
Prevalence in FD arm		0.39	0.15	0.06	0.04
Prevalence in HD arm		0.47	0.23	0.12	0.08
Good decision ^[3]^		U	U	U	U

HD arm: half-dose arm; FD arm: full-dose arm

^1^IVH: Intraventricular haemorrhage

^2^NEC: Necrotizing enterocolitis

^3^Good decision= What have been considered as good decision for each scenario and event: “Acc” if the difference of prevalence of events is *Acceptable*, “U” if the difference is *Unacceptable*

^4^Weight = Relative severity of the event according to the experts

^5^Class *a* misclassifications rate: Trials that conclude that the difference between arms is *Unacceptable*, among trials with acceptable difference

^6^Class *b* misclassifications rate: Trials that conclude that the difference between arms is *Acceptable*, among trials with unacceptable difference

The observations of each trial were sampled in *L* interim analyses. At each analysis, the analysis’ population will include the patients of the actual analysis and the patients of the *l*−1 previous analyses.

To address the issues of how prior location and precision may affect posterior inferences, we constructed an array of *P* alternative priors, each obtained by specifying numerical values of two quantities, one that changes the prior’s location *E*(*π*_1,*j*_−*π*_0,*j*_) and one that changes its precision (see more details in Section 2 of the Additional file 7).

#### Choice of the prior and thresholds for the final analysis

The posterior distributions of *θ*_1,*j*_−*θ*_0,*j*_ of the final *L*^*th*^ analysis, were obtained through the Hamiltonian-Monte Carlo method, using the rstan package [20, 21] carried out in R among the 5000 simulated trials. The posterior probability that it is higher than the acceptable difference distribution was calculated. Then, we calculated, the overall number of misclassifications obtained when applying the decision rule with different decision thresholds $\tau ^{l}_{j}$ at the final analysis, with $\tau ^{L}_{j} \in (0.50, 1.00)$. Considering the contingency table presented below, we defined two types of misclassifications:

**Table:** 

		Truth	
		The difference is Acceptable	The difference is Unacceptable
Conclusion of the decision rule	The difference is Unacceptable	A = Class *a* misclassification	D
	The difference is Acceptable	C	B = Class *b* misclassification

The rates of class *a* and class *b* misclassifications are =*A*/(*A*+*C*) and =*B*/(*B*+*D*), respectively.

This work was repeated for each event, using the *P* priors. Then, the most appropriate prior was selected, along with the thresholds $\tau ^{l}_{j}$ for each event, that is those that gave acceptable rates of class *a* and *b* misclassifications. Let *p*∗ denote the selected prior and $\tau *^{L}_{j}$ the selected decision thresholds at the L analysis for the event j.

#### Choice of the thresholds for the interim analyses

To construct the decision rule to be applied at each previous interim analysis, the simulation has been repeated for the *L* interim analyses, using the *p*∗ elected prior. The decision thresholds $\tau ^{l}_{j}$ were defined as follows: (i) for the final analysis, $\tau *^{L}_{j}$ was the one defined through the previous step, (ii) for the first analysis, $\tau *^{1}_{j}$ has been set to 0.95, (iii) for *l*∈(2,*L*−1), four decreasing functions have been tested to define $\tau ^{l}_{j}$ (see Table 3). The overall number of misclassifications obtained with those different functions has been compared. Then, the most appropriate function and thresholds $\tau *^{L}_{j}$ have been selected.

**Table 3:** Four functions applied to define the thresholds at each of the interim analyses

1	A uniform function: $\tau ^{l}_{j} = \tau *^{11}_{j}$ for all *l*∈(1,11).
2	A linear function: $\tau ^{l}_{j} = a \times l + b$,
3	A linear function with an exponential transformation: $\tau ^{l}_{j} = a \times exp^{l} + b$,
4	A linear function with a logarithm transformation: $\tau ^{l}_{j} = a \times log(l) + b$.

## Results

### Fit margins from experts’ elicitation

Among the 78 experts to which the questionnaire was sent, 44 answered (56.4%) (Table 4), including 43 who provided answers about acceptable rates of events in the half-dose arm.

**Table 4:** Main characteristics of the experts who answered to the elicitation questionnaire

Characteristics	N = 44
Age, median (IQR)	46 (39.75-55)
Male sex, n(%)	28 (64)
Number year of being MD ^[1]^, median (IQR)	17 (0.75-22.5)
Specialty, n(%)	
Neonatologist	22 (50)
Obstetrician	22 (50)
Type of establishment, n(%)	
University hospital	33 (75)
Position, n(%)	
Hospital practitioner	21 (48)
Professor	21 (48)
Others	2 (4)
History of school of statistics/epidemiology, n(%)	28 (64)
History of being PI ^[2]^ of a trial, n(%)	29 (66)

IQR = Interquartile Range

^1^MD: medical doctor

^2^PI: Principal investigator

**Fig. 2:**
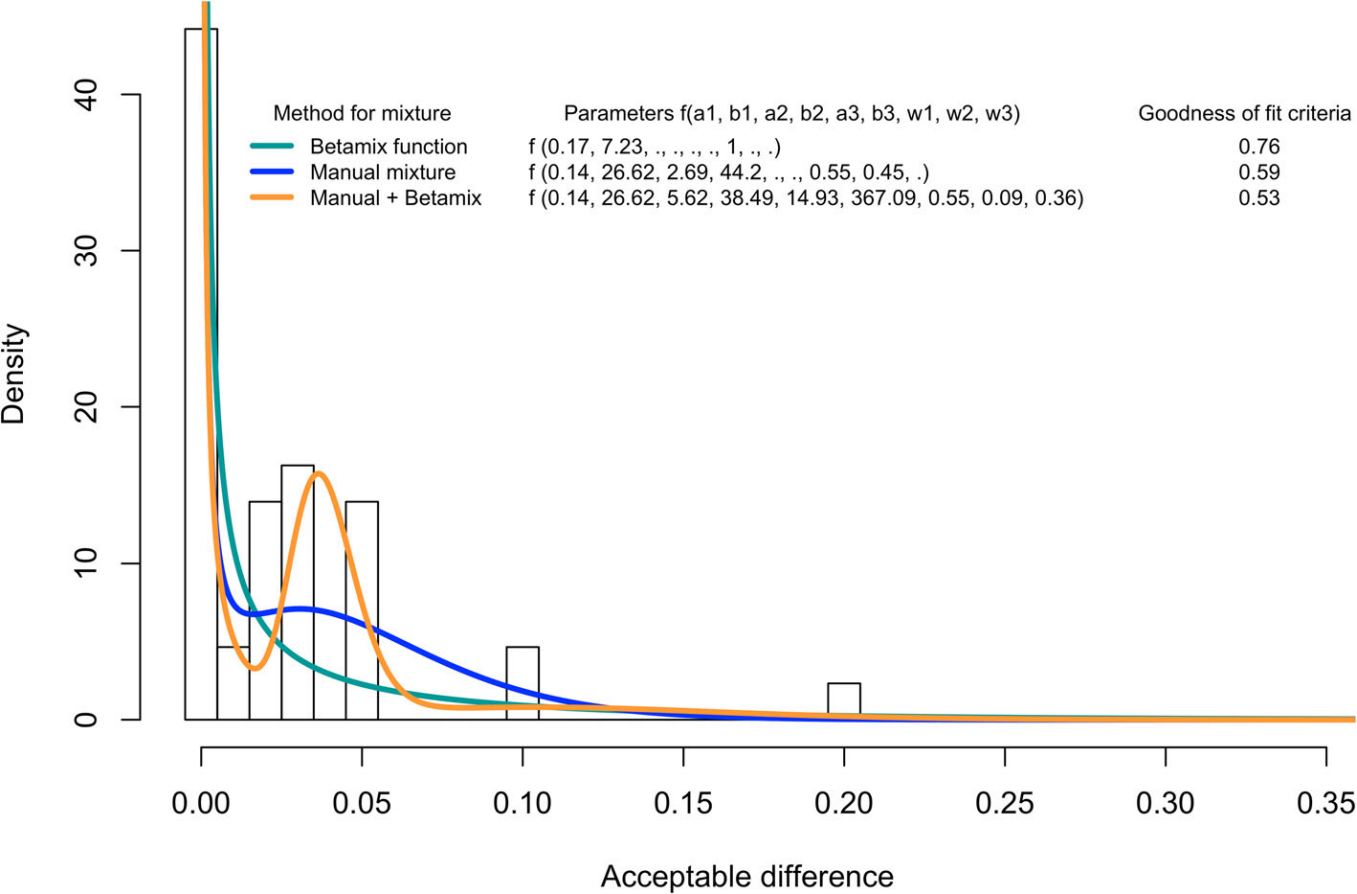
Histogram of the acceptable difference of severe intraventricular haemorrhage between arms, and mixtures of Beta distributions fitted from experts’ elicitation, through 3 different methods, with their criteria for goodness of fit. The histogram represents the acceptable difference of IVH among the *E* experts (*d*_*j,e*_). The 3 lines represent the fits of this difference (*D*_*j*_), obtained through the 3 different methods. The legend gives the parameters of the fits and their criteria for goodness of fit

Figure 2 presents the histogram of the acceptable differences of IVH among the *E* experts (*d*_*j,e*_), the fits (*D*_*j*_) obtained through the 3 different methods, and their criteria for goodness of fit. For the other events, see Additional file 2. The Additional file 3 summarizes the mixtures retained for the acceptable differences *D*_*j*_.

### Sensitivity analysis to select the prior and the decision thresholds

A good sequential decision rule is supposed to help in making a good decision, that is to advise when to stop the trial when the prevalence of events is truly unacceptable and to not stop when the difference is acceptable. Table 2 summarizes what was considered as a “good decision” according to each scenario and events (see more details in the Section 3 of the Additional file 7).

The maximum rate of class *a* misclassifications has been set to 0.10. For class *b* misclassifications, we set a maximum inversely proportional to the weight of the event according to the experts (Table 2). Denote by *W*_*j*_ the median weight of the *j* event among the *E* experts (*W*_*j*_∈[0,100] and *W*_*death*_=100), the maximal rate of class *b* misclassifications has been set to: $\text {Max}(\text {class b misclassification})_{j} = 0.1 + 0.50 \times \frac {100 - W_{j}}{100}$.

#### Selection of the prior and thresholds for the final analysis

**Fig. 3:**
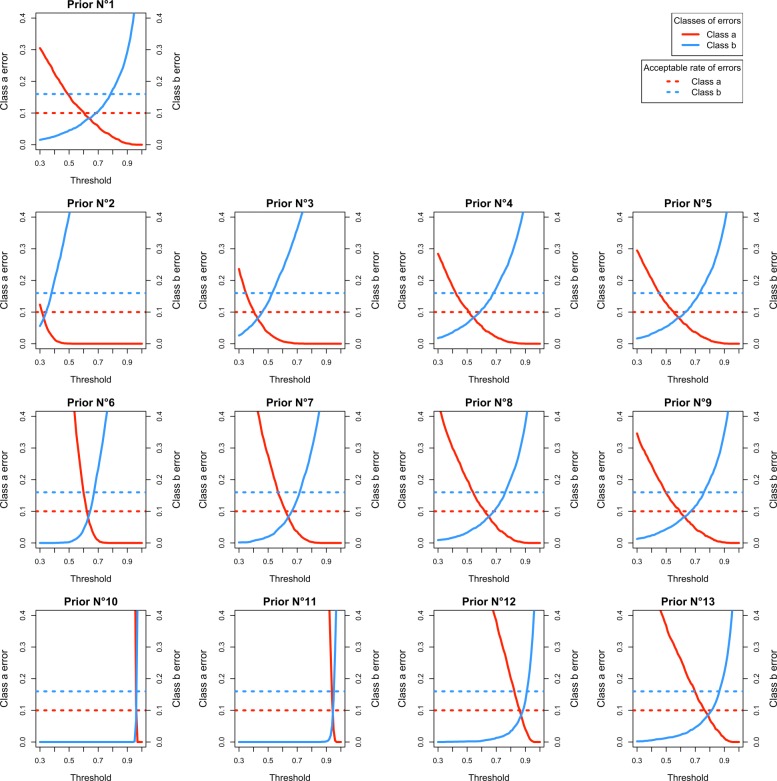
Plots of posterior class *a* and class *b* misclassifications according to the decision thresholds for each of the 13 pairs of priors for severe intraventricular haemorrhage. This figure represents the posterior rates of misclassifications for each pair of priors. Prior 1 is the non-informative prior, with *α*_1,*j*_=*α*_0,*j*_=*β*_1,*j*_=*β*_0,*j*_=1; Prior 2 to 13 are distinguished by (i) the means for the difference between the two arms: *E*(*π*_1,*j*_−*π*_0,*j*_)=0 for prior 2, 3, 4 and 5; *E*(*π*_1,*j*_−*π*_0,*j*_)=median(*d*_*j,e*_) for prior 6, 7, 8 and 9; and *E*(*π*_1,*j*_−*π*_0,*j*_)=*π*_0,*j*_ for prior 10, 11, 12 and 13; (ii) their precision: 1 for prior 2, 6 and 10; 1/3 for prior 3, 7 and 11; 1/10 for prior 4, 8 and 12; and 1/20 for prior 5, 9 and 13. For each prior, the red solid line represents the number of posterior class *a* misclassifications (trials that conclude that the difference between arms is *Unacceptable*, while it is not true) at the final analysis, according to each final threshold. The blue solid line represents the number of posterior class *b* misclassifications (trials that conclude that the difference between arms is *Acceptable*, while it is not true)

Figure 3 shows the number of posterior misclassifications at the final analysis according to each prior and final threshold for IVH. See Additional file 4 for the other events. In an effort to construct a homogeneous decision rule, we selected the same prior for all of the events. Several priors gave acceptable rates of misclassifications (prior 1, 3, 4, 5, 8, 9 and 13). We arbitrarily chose the prior Number 9. Conversely, we applied different final thresholds $\tau *^{L}_{j}$ for each event, as they are influenced by the prevalence of events and by the acceptable difference $\delta ^{l}_{j}$ (Table 5).

**Table 5:** Final decision rule retained through the sensitivity analysis: thresholds to be applied at each interim analysis and final overall rates of misclassifications, according to the event

	Event			
	Death	IVH ^[2]^	NEC ^[3]^	Retinopathy
Thresholds $\tau *^{L}_{j}$^[1]^, to applied in analysis				
1	0.95	0.95	0.95	0.95
2	0.95	0.95	0.95	0.95
3	0.95	0.95	0.95	0.95
4	0.95	0.95	0.95	0.95
5	0.949	0.949	0.950	0.949
6	0.948	0.948	0.949	0.948
7	0.944	0.944	0.948	0.946
8	0.934	0.933	0.944	0.938
9	0.907	0.903	0.932	0.918
10	0.832	0.821	0.902	0.862
11	0.63	0.60	0.82	0.71
Overall rate of errors				
Class *a* misclassifications ^[4]^	0.09	0.09	0.10	0.10
Class *b* misclassifications ^[5]^	<0.01	0.07	0.20	0.27

^1^$\tau *^{L}_{j}$ : threshold to apply in the Bayesian decision rule for the event *k* in the subgroup *j*, at the *l* interim analysis: the rule will conclude that their is an unacceptable excess if $ {P (\delta ^{l}_{j}) \geq \tau ^{l}_{j}}$

^2^IVH: Intraventricular haemorrhage

^3^NEC: Necrotizing enterocolitis

^4^Class *a* misclassifications: Trials that conclude that the difference between arms is *Unacceptable*, while it is not true

^5^Class *b* misclassifications: Trials that conclude that the difference between arms is *Acceptable*, while it is not true

#### Selection of the thresholds for the interim analyses

In our case-study, we set *L*=11. The number of misclassifications obtained by applying the 4 functions defining $\tau ^{l}_{j}$ are presented in the Additional file 5. We retained the linear function with an exponential transformation because it maintained the overall rate of misclassifications under the prespecified acceptable rates. The 3 other functions increased the rate of class *a* misclassifications over 0.10.

**Fig. 4:**
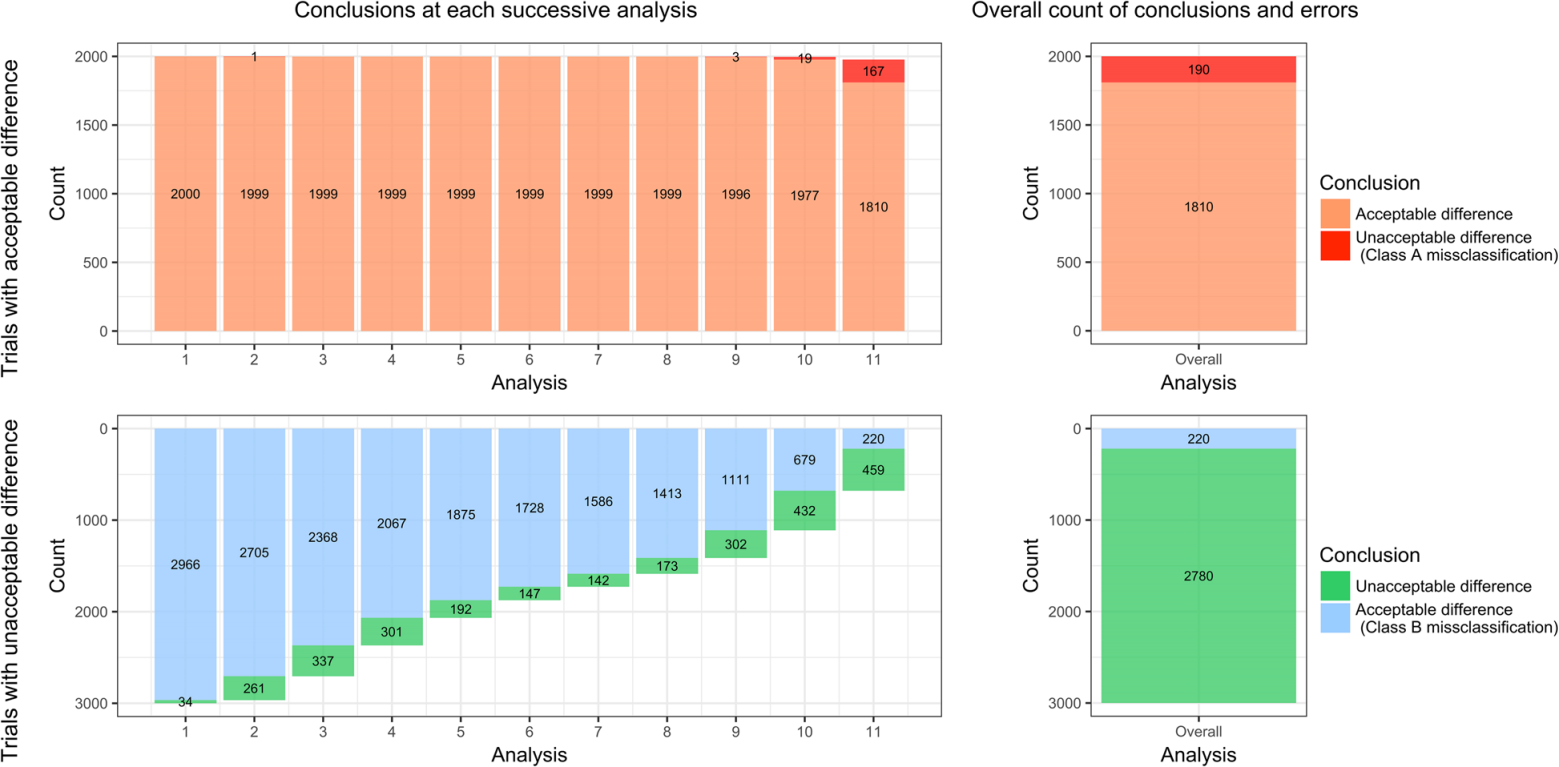
Distribution of the successive conclusions and errors, obtained by applying the decision rule to the 5000 simulated trials, at each interim analysis and in overall, for severe intraventricular haemorrhage. The left part of the plot represents the conclusions at each interim analysis. The right part represents the overall count of conclusions among the 11 analyses. The upper part of the plot represents the trials with an *Acceptable* difference between arms: orange area correspond to trials that conclude that the difference between arms is *Acceptable*, while it is true; red area correspond to trials that conclude that the difference between arms is *Unacceptable*, while it is not true (class *a* misclassifications). The bottom part of the plot represents the trials with an *Unacceptable* difference between arms: green area correspond to trials that conclude that the difference between arms is *Unacceptable*, while it is true; blue area correspond to trials that conclude that the difference between arms is *Acceptable*, while it is not true (class *b* misclassifications)

**Fig. 5:**
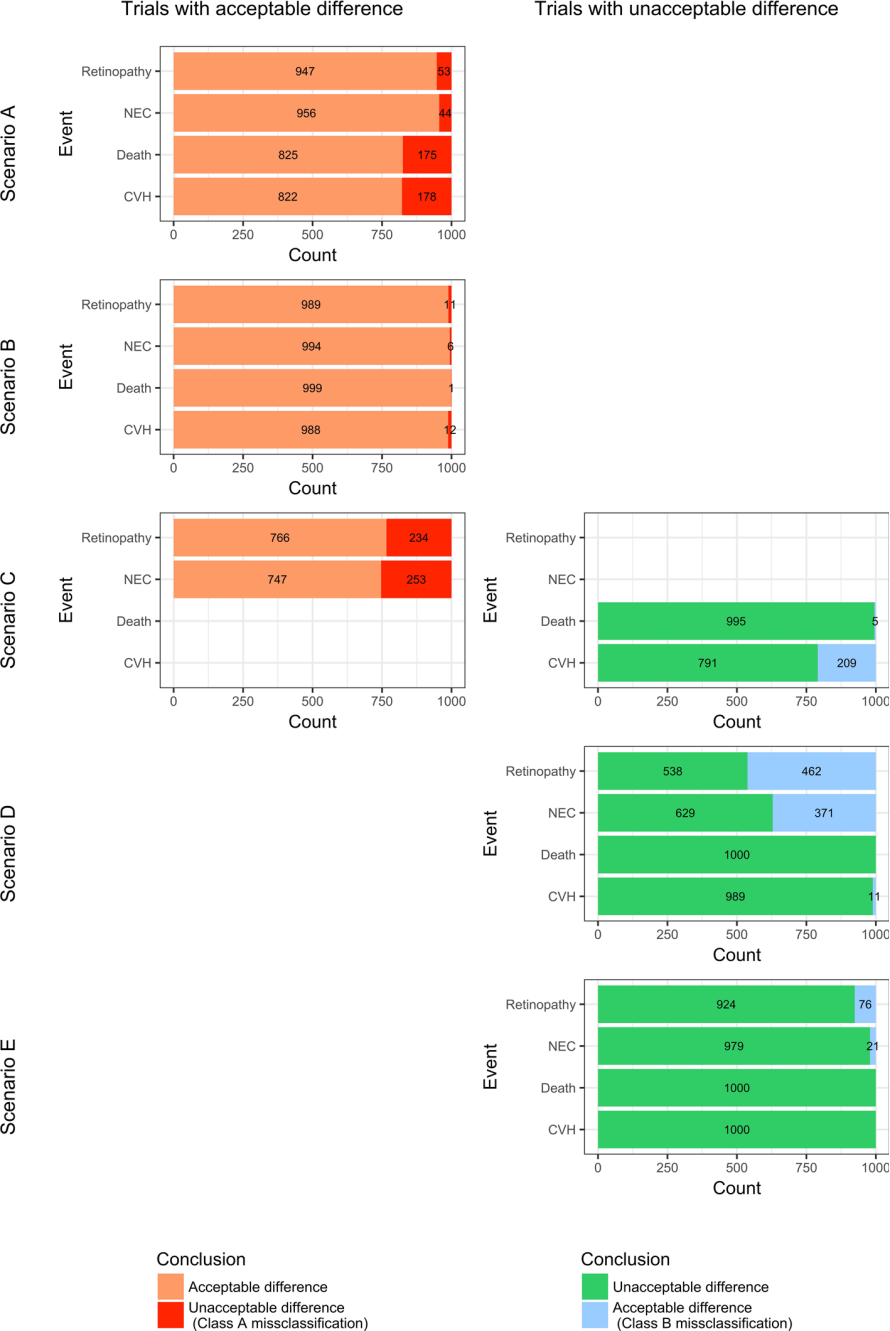
Distribution of the overall conclusions and errors, obtained by applying the decision rule to the 5000 simulated trials, according to the event and the scenario. This plot presents the overall numbers or misclassifications obtained by applying this decision rule, according to the 5 scenario and to the 4 events. The left part of the plot represents the trials with an *Acceptable* difference between arms: orange area correspond to trials that conclude that the difference between arms is *Acceptable*, while it is true; red area correspond to trials that conclude that the difference between arms is *Unacceptable*, while it is not true (class *a* misclassifications). The right part of the plot represents the trials with an *Unacceptable* difference between arms: green area correspond to trials that conclude that the difference between arms is *Unacceptable*, while it is true; blue area correspond to trials that conclude that the difference between arms is *Acceptable*, while it is not true (class *b* misclassifications). IVH: Intraventricular haemorrhage; NEC: Necrotizing enterocolitis

Table 5 summarizes the thresholds finally retained in the decision rule, $\tau *^{L}_{j}$, and the overall rates of misclassifications. Figure 4 gives the distribution of the conclusions and misclassifications among the trials, at each interim analysis and in total, for IVH. Additional file 6 represents the distribution of the conclusions and misclassifications for the other events. Finally, Fig. 5 presents the overall numbers or misclassifications obtained by applying this decision rule, according to the scenario.

### Application to data

We applied our method to a simulated dataset for the BETADOSE trial. In this dataset, we considered that the final sample size was 1571 per arm, with *n*_*i*_=162 for children born before 28 weeks. The prevalence of events was sampled as detailed in Table 2. For all events, a good decision of this trial was considered to conclude an “Unacceptable” difference using the same explanation given before (Section 3 of the Additional file 7). Table 6 summarizes the results at the end of the trial (expressed as observed prevalence) and the Bayesian sequential results, using the rule built in the previous step (Table 5).

**Table 6:** Observations and decision obtained by application of the Bayesian decision rule to a data set

Analysis	N		Death				Intraventricular haemorrhage						Necrotising enterocolitis						Retinopathy							
			Observations				Bayesian		Observations				Bayesian		Observations				Bayesian		Observations				Bayesian	
	FD	HD	FD		HD		Post	Deci-	FD		HD		Post	Deci-	FD		HD		Post	Deci-	FD		HD		Post	Deci-
	arm	arm	arm		arm		prob.	sion	arm		arm		prob.	sion	arm		arm		prob.	sion	arm		arm		prob.	sion
			n	%	n	%			n	%	n	%			n	%	n	%			n	%	n	%		
1	10	20	5	0.50	11	0.55	0.60	0	0	0	1	0.05	0.42	0	0	0	1	0.05	0.59	0	0	0	2	0.10	0.69	0
2	25	35	12	0.48	20	0.57	0.66	0	1	0.04	6	0.17	0.71	0	1	0.04	2	0.06	0.55	0	0	0	4	0.11	0.84	0
3	39	51	16	0.41	29	0.57	0.79	0	3	0.08	9	0.18	0.70	0	3	0.08	7	0.14	0.75	0	0	0	5	0.10	0.87	0
4	54	68	22	0.41	42	0.62	0.89	0	4	0.07	13	0.19	0.80	0	5	0.09	10	0.15	0.74	0	0	0	8	0.12	0.95	0
5	77	80	32	0.42	53	0.66	0.94	0	8	0.10	17	0.21	0.81	0	5	0.06	10	0.12	0.79	0	4	0.05	9	0.11	0.78	0
6	88	92	34	0.39	58	0.63	0.95	1	9	0.10	22	0.24	0.89	0	6	0.07	12	0.13	0.81	0	5	0.06	10	0.11	0.76	0
7	99	104	39	0.39	65	0.62	0.95		14	0.14	25	0.24	0.81	0	10	0.10	16	0.15	0.76	0	5	0.05	12	0.12	0.83	0
8	116	121	45	0.39	72	0.60	0.94		16	0.14	31	0.26	0.88	0	10	0.09	18	0.15	0.83	0	6	0.05	12	0.10	0.76	0
9	127	139	50	0.39	80	0.58	0.93		18	0.14	33	0.24	0.84	0	11	0.09	20	0.14	0.83	0	6	0.05	13	0.09	0.76	0
10	143	157	54	0.38	86	0.55	0.93		21	0.15	37	0.24	0.83	1	12	0.08	24	0.15	0.88	0	6	0.04	15	0.10	0.82	0
11	154	163	60	0.39	90	0.55	0.92		24	0.16	37	0.23	0.76		12	0.08	24	0.15	0.89	1	7	0.05	15	0.09	0.78	1

HD arm: half-dose arm; FD arm: full-dose arm

At the 6^*th*^ analysis, since the posterior probabilities became higher than the prespecified threshold $\tau *^{6}_{j}$ for death, the trial was stopped because of a potential unacceptable increase of deaths in the experimental arm. If the trial had continued, it would have stopped at the 10^*th*^ analysis because of IVH.

## Discussion

Motivated by the desire to deal with settings where rare but serious events have to be monitored during an non-inferiority trial, we have proposed a methodology that provides a practical way to help in the decision making at each interim analysis.

Our approach has the advantage of incorporating experts’ opinions about the non-inferiority margins. As a consequence, it can be used as an alternative in cases where historical placebo-controlled data aren’t available. We have proposed to keep the variability among experts and used a distribution instead of a discrete margin. Indeed, we could have averaged all experts’ opinions, but this will not have reflected all potential variability. In a simulation study, we compared our approach to the use of average values (see Additional file 9). We found that the use of a mixture gave different results than the use of the mean of the experts’ opinions. Indeed, the difference between the two approaches increased as the variability among experts increased. Moreover, we could have weighted experts’ opinions according to some pertinent covariates. In a previous work, Thall et al. compared different ways to weight physicians’ opinion using mixture priors of the parameter of interest [22]. The authors found, according to their design, that posterior quantities appear to be insensitive to how physicians are weighted, so we decided to weight all physicians equally. In our case, the variability among experts was kept in order to reflect all potential opinions, that is the distribution across all the range of potential margins. Our method can be applied whatever the values are in between zero and one.

One limitation of our motivating example is that the majority of the experts set the acceptable difference to zero, whereas zero is not a possible value for a non-inferiority margin. When generalizing this method to another non-inferiority trial, we suggest to investigators to remind the experts that the margin cannot be set to zero.

Because the prior chosen for a Bayesian analysis needs to be well documented and robust to its parameter choices, we performed an extensive sensitivity analysis evaluating non-informative and informative priors and several thresholds. Thresholds retained were varying between events, allowing us to consider the differences in prevalence, and in margins and severity conferred by clinicians to each event. Likewise, when we repeated this work in the subgroup of premature infants born after 28 weeks (results not shown), the thresholds were different, reflecting the higher rarity of events and the different margins.

To choose the best priors and stopping thresholds, the rates of misclassifications have been computed and compared. As the two types of misclassifications are moving in opposite directions, we had to find a compromise between the two. Since we do not want to wrongly conclude too often an inferiority of the experimental arm, we decided to set a maximum for class A misclassification at 0.10, to be more permissive in terms of class B misclassifications and to adapt this permissiveness to the severity of each event. To define the stopping thresholds at each interim analysis, simulations have compared several initial thresholds and four decreasing functions of *τ*. The purpose of this study was to find the best thresholds in order to have good functional properties of the design, i.e. do not stop frequently at the beginning when it is wrong and do not continue until the end when we have to stop. Finally, as we dealt with some rare events, overall rates of class A and B misclassifications were relatively high. This has to be put in balance with frequentist type I and type II error rates that sometimes have to be compromised, especially in the case of rare secondary events.

When generalizing this method to another trial, this work needs to be repeated before the analysis of the real data; the maximal rates of class A and B misclassifications have to be balanced, considering the setting, and the parameters of the decision rule have to be adapted in consequence, namely the prior, the margins and the decision thresholds. Finally, after having pre-specified all these parameters, the decision rule can be applied by the statistician to the unblinded data, and presented to the Data Safety Monitoring Board. In order to apply this methodology, we already designed a non-inferiority trial that should start in few months, using the same statistical approach in an other setting.

In conclusion, our approach was found to be efficient in dealing with safety monitoring of rare and non-rare events in a non-inferiority context. It requires a strong collaboration between physicians and the trial statisticians for the benefit of all.

## Conclusion

We proposed a practical way to help to assist with decisions on safety dichotomous events at each interim analysis of a non-inferiority trial. This Bayesian design is suitable for rare events and for non-rare events. It incorporates experts’ opinions on margins, so it can be constructed without historical placebo-controlled data. This Bayesian sequential approach could be applied as a complement to the frequentist analysis, so both Data Safety Monitoring Boards and investigators can benefit from such an approach.

## Additional files

Additional file 1 Expected distribution of gestational age and events in the control arm (FD arm) of the BETADOSE trial, imputed from the prevalence observed in the ePIPAGE-2 cohort study. A table providing the expected event rates and gestational age in the motivated trial (based on the EPIPAGE-2 cohort study [17]). (PDF 78 kb)

Additional file 2 Histogram of the acceptable differences in events, and mixtures of beta distributions fitted from experts’ elicitation, through 3 different methods, with their criteria for goodness of fit. The plots analogous to Fig. 2 for the 3 other events: (a) Death, (b) Necrotizing enterocolitis, (c) Retinopathy. (PDF 992 kb)

Additional file 3 Parameters of the mixtures retained to fit the acceptable differences between arms *D*_*j*_. A table summarizing the mixtures retained for the acceptable differences. (PDF 108 kb)

Additional file 4 Plots of posterior class *a* and class *b* misclassifications according to the stopping thresholds for each of the 13 pairs of priors. The plots analogous to Fig. 3 for the 3 other events: (a) Death, (b) Necrotizing enterocolitis, (c) Retinopathy. (PDF 2969 kb)

Additional file 5 Final overall rates of misclassifications obtained with the decision rule, according to the function used to define the thresholds at each successive interim analysis. The final overall rates of misclassifications obtained with the decision rule summarized in the Table 5, provided for the 4 functions used to define the thresholds at each successive interim analysis. (PDF 107 kb)

Additional file 6 Distribution of the successive conclusions and misclassifications, obtained by applying the decision rule to the 5000 simulated trials at each interim analysis and in overall. The plots analogous to Fig. 4 for the 3 other events: (a) Death, (b) Necrotizing enterocolitis, (c) Retinopathy. (PDF 2498 kb)

Additional file 7 Supplemental information’s on methods. Additional information on methods : (i) Method for comparison of the 3 different estimation ways used to fit the mixtures of Beta distributions; (ii) Definition of the non-informative and informative priors compared in the sensitivity analysis; (iii) Definition of the “good decision” for each scenario of the simulation study. (PDF 246 kb)

Additional file 8 Physician experts who participated in the elicitation, BETADOSE trial. Complete list of experts who participated in the elicitation. (PDF 41 kb)

Additional file 9 Comparison of the use of a mixture distribution versus the use of an average value of elicitated margins from experts. In this additional work, two approaches were proposed to compute the acceptable difference from experts’ elicitation: the method described in the main manuscript and the use of average values. (PDF 368 kb)

Additional file 10 Example of the code to simulate the results for one event (neonatal death): In the part A, it fit margins from experts’ elicitation, using a fictive data set of experts’ answers (E1). In the part B, it simulate the trials and compute the differences of the posterior samplers for M pairs, for one scenario, using one informative prior. In the part C, it calculate the posterior probability that the difference of rate of events is higher than the acceptable difference according to experts, and compute the decision using a threshold of 0.50. (R 26 kb)

## Data Availability

We provided as a supplementary material an example of the code to run the 2 steps of this work (Additional file 10).The complete materials used for this study (R code and dataset generated) are available from the corresponding author on reasonable request.

## Abbreviations

IVH: Intraventricular haemorrhage
NI: Non-inferiority
