# Supplementary material for: A Bayesian non-inferiority approach using experts’ margin elicitation – application to the monitoring of safety events
Source: BMC Med Res Methodol. 2019 Sep 18;19:187. doi: 10.1186/s12874-019-0826-5 (PMC6751616; doi:10.1186/s12874-019-0826-5)
Supplement: Supplementary file 1 — Expected distribution of gestational age and events in the control arm (FD arm) of the BETADOSE trial, imputed from the prevalence observed in the ePIPAGE-2 cohort study. A table providing the expected event rates and gestational age in the motivated trial (based on the EPIPAGE-2 cohort study [17]). (PDF 78 kb) [file 12874_2019_826_MOESM1_ESM.pdf]

Expected distribution of gestational age and events in the control arm (FD arm) of the BETADOSE trial, imputed from the prevalence observed in the EPIPAGE-2 cohort study. [1]

**Gestational age at birth**	**Expected number of births in the FD arm**[2]	**Expected number of events**			
		**Death**	**IVH** [3]	**NEC** [4]	**Retino-pathy**
	N	% (n)	% (n)	% (n)	% (n)
<28 weeks	162	39% (63)	15% (24)	6% (10)	4% (6)
28-32 weeks	524	5% (26)	3% (16)	3% (16)	<0.1% (<1)
$\geq$ 32 weeks	887	<1% (<9)	<1% (<9)	<1% (<9)	<0.1% (<1)
Total	1574	<6% (<98)	<3% (<49)	<2% (<35)	<1% (<8)

[1]Pierre-Yves Ancel, Franois Goffinet, EPIPAGE-2 Writing Group, et al. Survival and morbidity of preterm children born at 22 through 34 weeks gestation in France in 2011: results of the EPIPAGE-2 cohort study. JAMA pediatrics, 169(3):230238, March 2015.

[2]FD arm: full-dose arm

[3]IVH: Intraventricular hemorrhage

[4]NEC: Necrotizing enterocolitis
