# Supplementary material for: A Bayesian non-inferiority approach using experts’ margin elicitation – application to the monitoring of safety events
Source: BMC Med Res Methodol. 2019 Sep 18;19:187. doi: 10.1186/s12874-019-0826-5 (PMC6751616; doi:10.1186/s12874-019-0826-5)
Supplement: Supplementary file 2 — Histogram of the acceptable differences in events, and mixtures of beta distributions fitted from experts’ elicitation, through 3 different methods, with their criteria for goodness of fit. The plots analogous to Fig. 2 for the 3 other events: (a) Death, (b) Necrotizing enterocolitis, (c) Retinopathy. (PDF 992 kb) [file 12874_2019_826_MOESM2_ESM.pdf]

Histogram of the acceptable differences in events, and mixtures of Beta distributions fitted from experts' elicitation, through 3 different methods, with their criteria for goodness of fit.

(a) Death

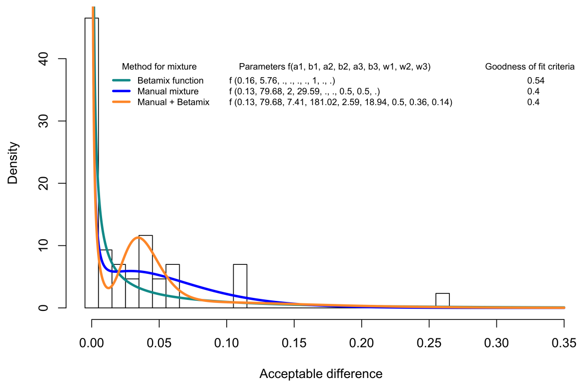

(b) Necrotising enterocolitis

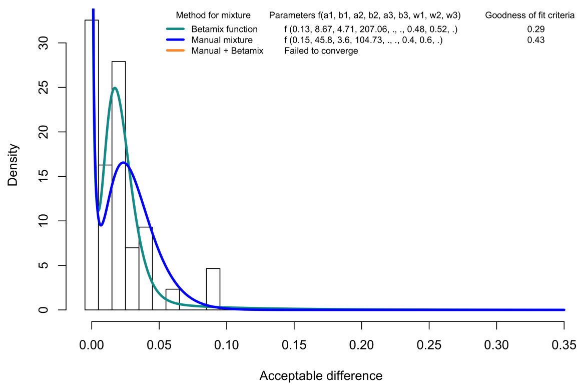

(c) Retinopathy

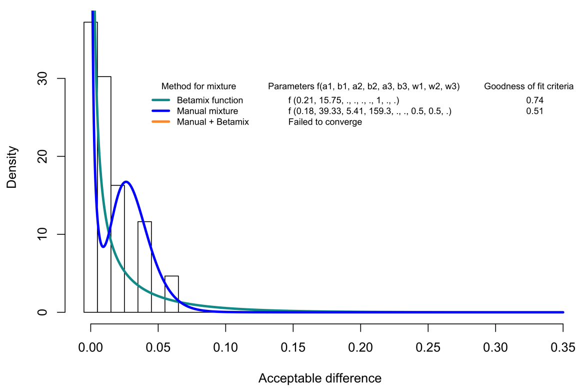

To fit the acceptable difference between arms D_j as mixtures of beta regression using maximum likelihood estimation through the `betareg` package on `R` software, 3 different estimation methods were adopted and compared using a criteria for goodness of fit based on area under curves (See Box 1 in the main manuscript and section 1 of the Web-Appendix C for details): (the first (`betamix` function) mathematically driven and the other two (Manual mixture; Manual + `betamix` function) empirically driven): the first (`betamix` function) mathematically driven and the other two (Manual mixture; Manual + `betamix` function) empirically driven.
