# Supplementary material for: A Bayesian non-inferiority approach using experts’ margin elicitation – application to the monitoring of safety events
Source: BMC Med Res Methodol. 2019 Sep 18;19:187. doi: 10.1186/s12874-019-0826-5 (PMC6751616; doi:10.1186/s12874-019-0826-5)
Supplement: Supplementary file 3 — Parameters of the mixtures retained to fit the acceptable differences between arms Dj. A table summarizing the mixtures retained for the acceptable differences. (PDF 108 kb) [file 12874_2019_826_MOESM3_ESM.pdf]

Parameters of the mixtures retained to fit the acceptable differences between arms D_j [1]

	**Event (j)**			
	**Death**	**IVH [2]**	**NEC [3]**	**Retinopathy**
**Method with the best criteria for goodness of fit [4]**	Manual mixture	Manual + Betamix	Betamix function	Manual mixture
**Parameters of the mixture [1]**				
$(a_{1,j}, b_{1,j})$	(0.13, 79.68)	(0.14, 26.62)	(4.71, 207.08)	(0.18, 39.33)
$w_{1,j}$	0.50	0.55	0.52	0.50
$(a_{2,j}, b_{2,j})$	(2, 29.59)	(5.62, 38.5)	(0.13, 8.67)	(5.41, 159.3)
$w_{2,j}$	0.50	0.09	0.48	0.50
$(a_{3,j}, b_{3,j})$	na	(14.93, 367.09)	na	na
$w_{3,j}$	na	0.36	na	na

na = not applicable

[1] D_j : Acceptable difference of event j between arms; the distribution of D_j is denoted $D_j \sim f(a_{1,j}, b_{1,j}, a_{2,j}, b_{2,j}, a_{3,j}, b_{3,j}, w_{1,j}, w_{2,j}, w_{3,j})$, where $(a_{1,j}, b_{1,j})$, $(a_{2,j}, b_{2,j})$ and $(a_{3,j}, b_{3,j})$ are parameters for the 3 *beta* distributions, and $(w_{1,j}, w_{2,j}, w_{3,j})$ the corresponding weights.

[2] IVH: Intraventricular hemorrhage

[3] NEC: Necrotizing enterocolitis

[4] To fit the acceptable difference between arms D_j as mixtures of beta regression using maximum likelihood estimation through the `betareg` package on `R` software, 3 different estimation methods were adopted and compared using a criteria for goodness of fit based on area under curves (See Box 1 in the main manuscript and section 1 of the Web-Appendix C for details): (the first (`betamix` function) mathematically driven and the other two (Manual mixture; Manual + `betamix` function) empirically driven): the first (`betamix` function) mathematically driven and the other two (Manual mixture; Manual + `betamix` function) empirically driven.
