# Supplementary material for: A Bayesian non-inferiority approach using experts’ margin elicitation – application to the monitoring of safety events
Source: BMC Med Res Methodol. 2019 Sep 18;19:187. doi: 10.1186/s12874-019-0826-5 (PMC6751616; doi:10.1186/s12874-019-0826-5)
Supplement: Supplementary file 4 — Plots of posterior class a and class b misclassifications according to the stopping thresholds for each of the 13 pairs of priors. The plots analogous to Fig. 3 for the 3 other events: (a) Death, (b) Necrotizing enterocolitis, (c) Retinopathy. (PDF 2969 kb) [file 12874_2019_826_MOESM4_ESM.pdf]

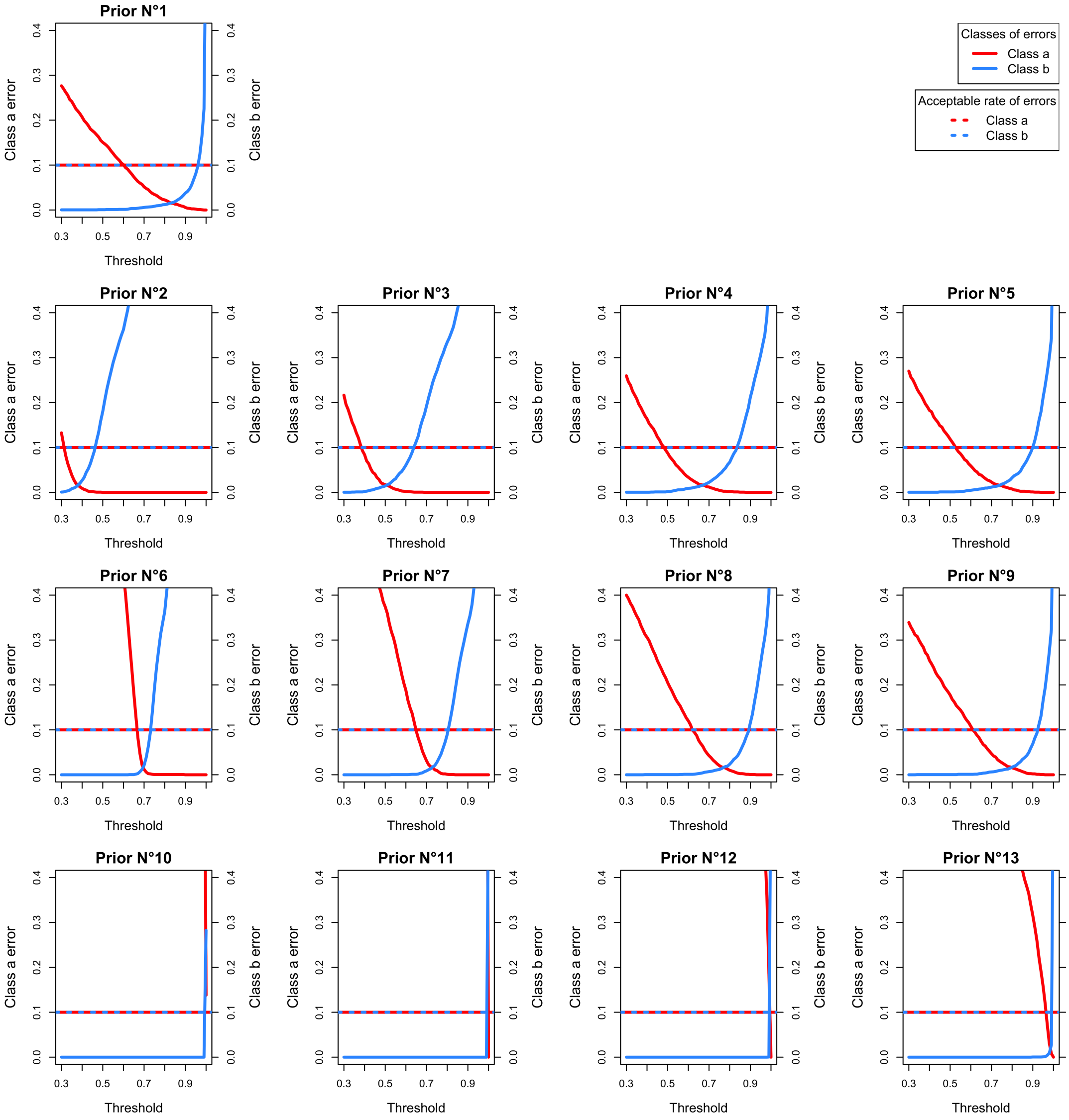

(a) Death

Plots of posterior class *a* and class *b* misclassifications according to the stopping thresholds for each of the 13 pairs of priors.

Prior 1: Non-informative prior, with $\alpha_{1,j} = \alpha_{0,j} = \beta_{1,j} = \beta_{0,j} = 1$; Prior 2 to 13 are distinguished by (i) the means for the difference between the two arms: $E(\pi_{1,j} - \pi_{0,j}) = 0$ for prior 2, 3, 4 and 5; $E(\pi_{1,j} - \pi_{0,j}) = \text{median}(d_{j,e})$ for prior 6, 7, 8 and 9; and $E(\pi_{1,j} - \pi_{0,j}) = \pi_{0,j}$ for prior 10, 11, 12 and 13; (ii) their precision: 1 for prior 2, 6 and 10; 1/3 for prior 3, 7 and 11; 1/10 for prior 4, 8 and 12; and 1/20 for prior 5, 9 and 13.

Class a misclassifications: Trials that conclude that the difference between arms is *Unacceptable*, while it is not true; Class b misclassifications: Trials that conclude that the difference between arms is *Acceptable*, while it is not true.

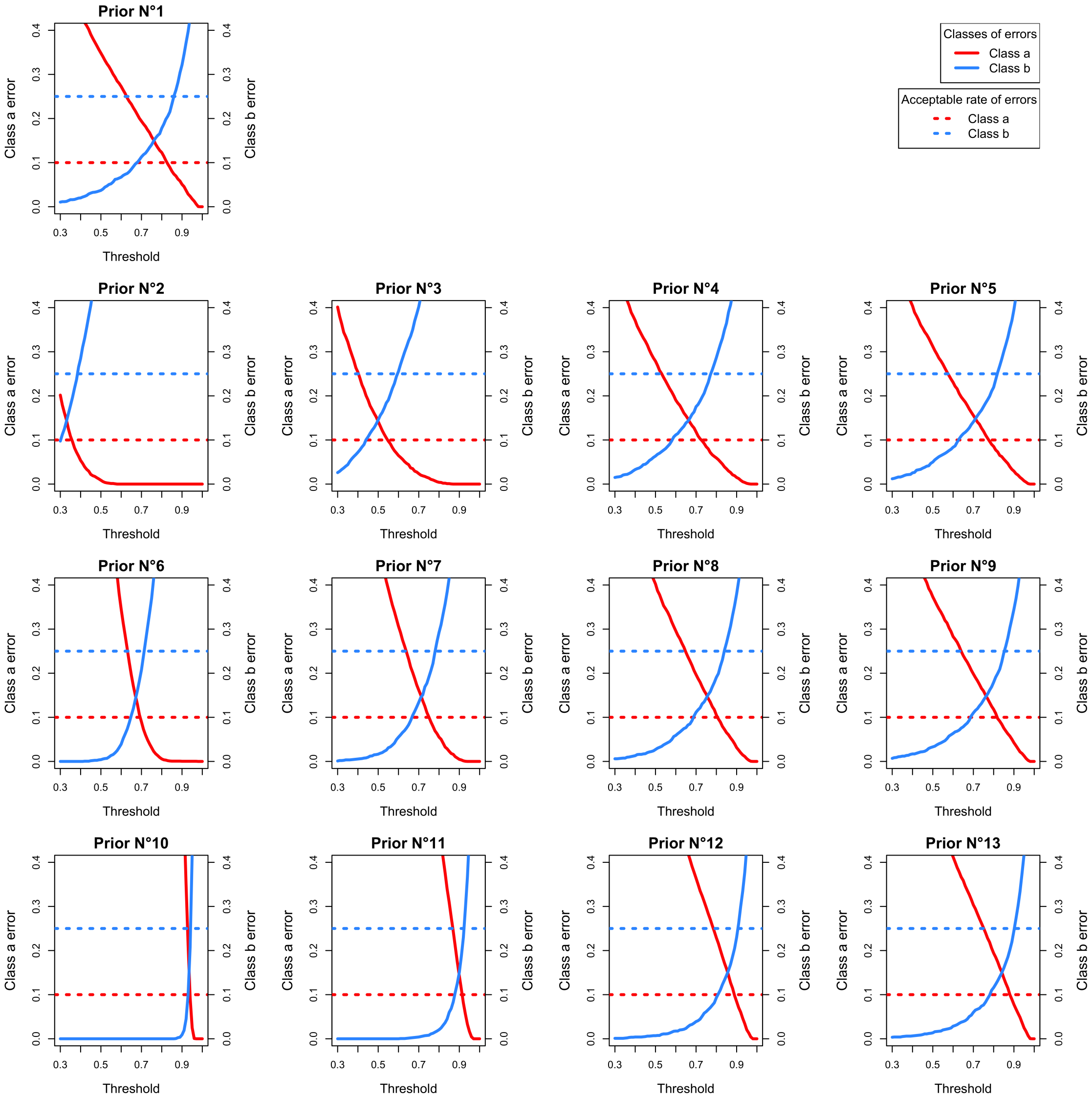

(b) Necrotizing enterocolitis

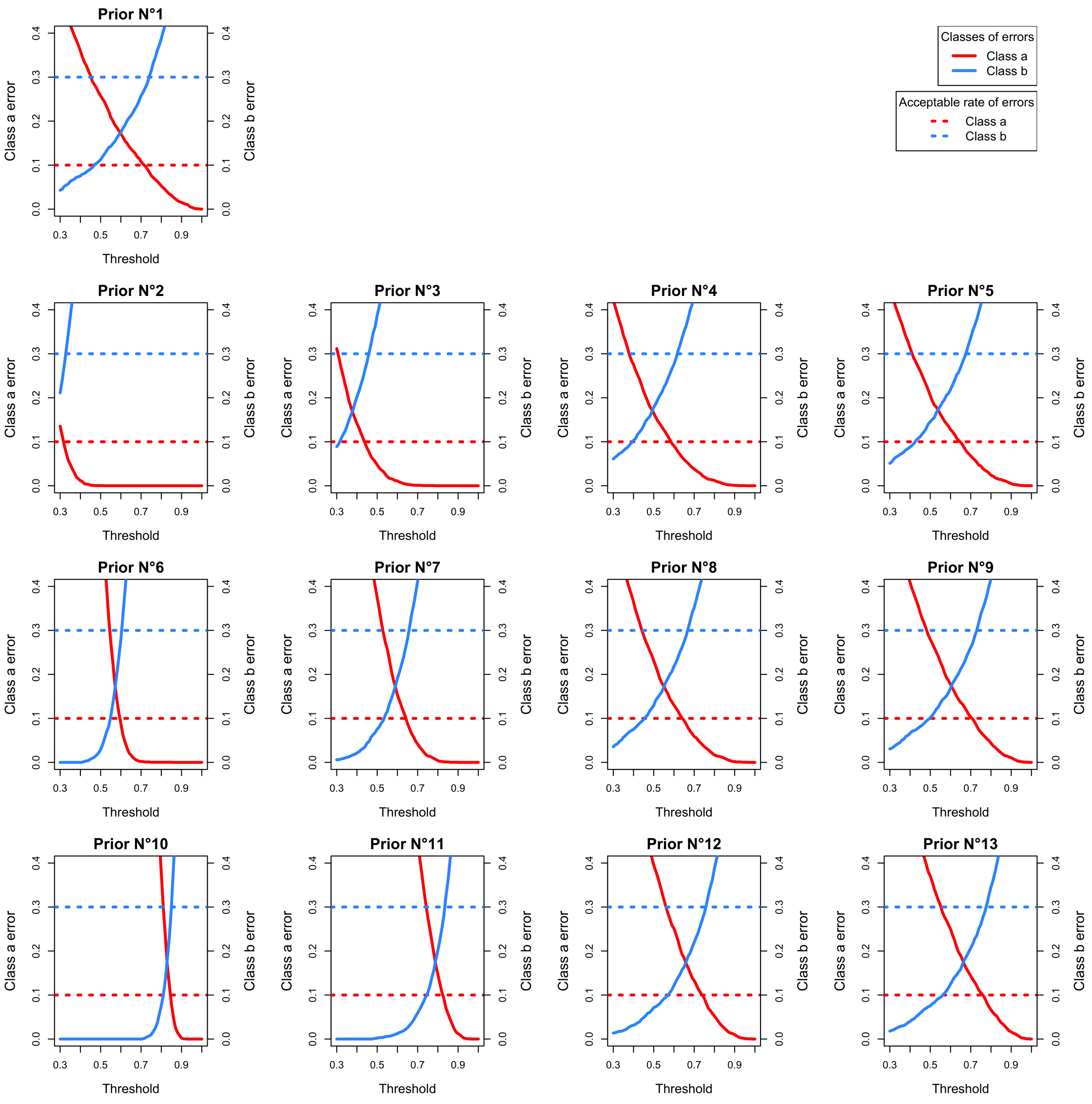

(c) Retinopathy
