# Supplementary material for: A Bayesian non-inferiority approach using experts’ margin elicitation – application to the monitoring of safety events
Source: BMC Med Res Methodol. 2019 Sep 18;19:187. doi: 10.1186/s12874-019-0826-5 (PMC6751616; doi:10.1186/s12874-019-0826-5)
Supplement: Supplementary file 5 — Final overall rates of misclassifications obtained with the decision rule, according to the function used to define the thresholds at each successive interim analysis. The final overall rates of misclassifications obtained with the decision rule summarized in the Table 5, provided for the 4 functions used to define the thresholds at each successive interim analysis. (PDF 107 kb) [file 12874_2019_826_MOESM5_ESM.pdf]

Final overall rates of misclassifications obtained with the decision rule, according to the function used to define the thresholds at each successive interim analysis.

Function applied to define the thresholds τ_j^l [1]	Class of misclassification [2]	Event			
		Death	IVH [3]	NEC [3]	Retinopathy
**Uniform** [1]	Class A	0.31	0.33	0.24	0.27
	Class B	<0.01	0.03	0.12	0.15
**Linear** [1]	Class A	0.11	0.12	0.14	0.12
	Class B	<0.01	0.06	0.17	0.23
**Linear with logarithm transformation** [1]	Class A	0.14	0.16	0.17	0.16
	Class B	<0.01	0.05	0.14	0.20
**Linear with exponential transformation** [1]	Class A	0.09	0.09	0.10	0.10
	Class B	<0.01	0.07	0.20	0.27

[1] τ_j^l : Thresholds to apply in the Bayesian decision rule for the event j, at the l interim analysis: the rule will conclude that their is an unacceptable excess if $P(\delta_j^l) \geq \tau_j^l$. Four functions have been compared: (i) A uniform function: $\tau_j^l = \tau*_j^{11}$ for all $l \in (1, 11)$; (ii) A linear function: $\tau_j^l = a \times l + b$;(iii) A linear function with a logarithm transformation: $\tau_j^l = a \times log(l) + b$; (iv) A linear function with an exponential transformation: $\tau_j^l = a \times exp^l + b$.

[2] Class a misclassification: Trials that conclude that the difference between arms is *Unacceptable*, while it is not true; Class b misclassification: Trials that conclude that the difference between arms is *Acceptable*, while it is not true.

[3] NEC: Necrotizing enterocolitis; IVH: Intraventricular hemorrhage
