# Supplementary material for: A Bayesian non-inferiority approach using experts’ margin elicitation – application to the monitoring of safety events
Source: BMC Med Res Methodol. 2019 Sep 18;19:187. doi: 10.1186/s12874-019-0826-5 (PMC6751616; doi:10.1186/s12874-019-0826-5)
Supplement: Supplementary file 6 — Distribution of the successive conclusions and misclassifications, obtained by applying the decision rule to the 5000 simulated trials at each interim analysis and in overall. The plots analogous to Fig. 4 for the 3 other events: (a) Death, (b) Necrotizing enterocolitis, (c) Retinopathy. (PDF 2498 kb) [file 12874_2019_826_MOESM6_ESM.pdf]

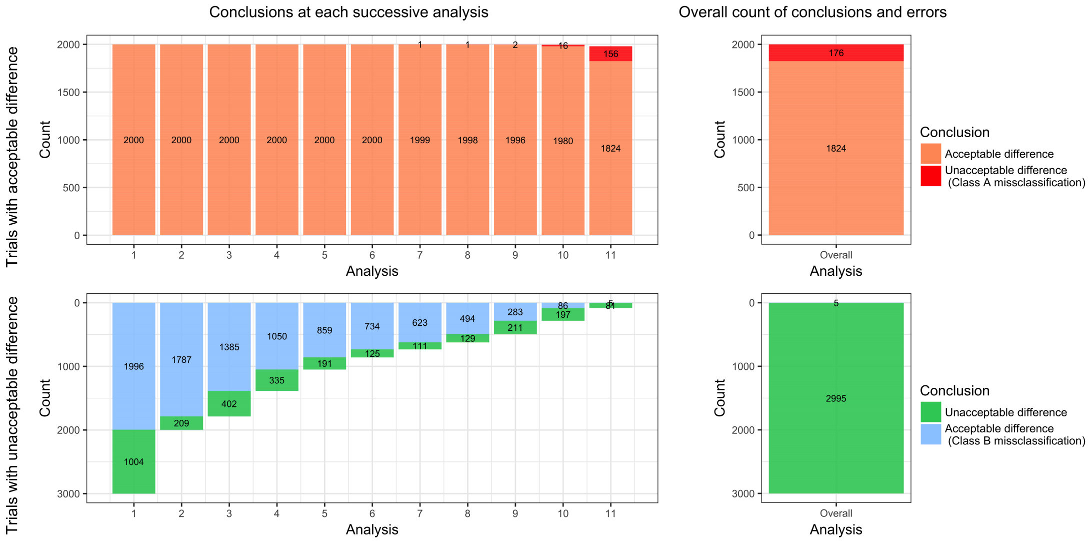

(a) Death

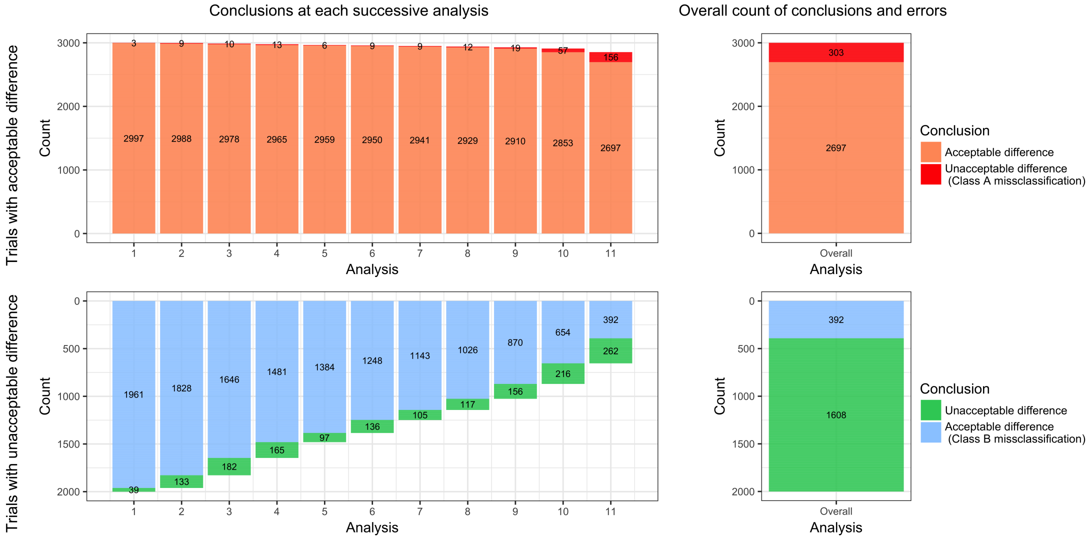

(b) Necrotizing enterocolitis

Distribution of the successive conclusions and misclassifications, obtained by applying the decision rule to the 5000 simulated trials at each interim analysis and in overall.

Class a misclassifications: Trials that conclude that the difference between arms is *Unacceptable*, while it is not true; Class b misclassifications: Trials that conclude that the difference between arms is *Acceptable*, while it is not true.

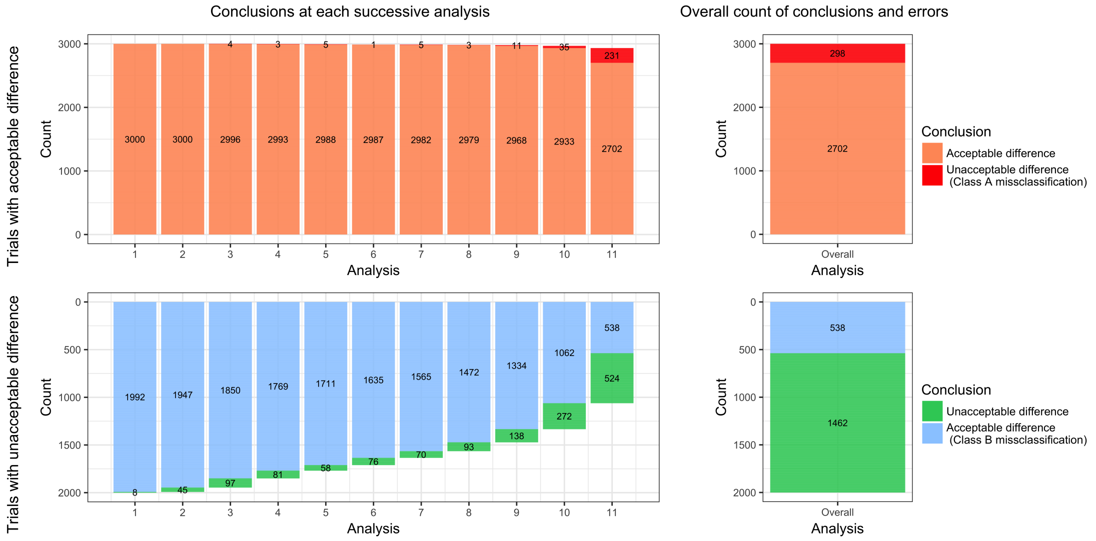

(c) Retinopathy

Class a misclassifications: Trials that conclude that the difference between arms is *Unacceptable*, while it is not true; Class b misclassifications: Trials that conclude that the difference between arms is *Acceptable*, while it is not true.
