# Supplementary material for: A Bayesian non-inferiority approach using experts’ margin elicitation – application to the monitoring of safety events
Source: BMC Med Res Methodol. 2019 Sep 18;19:187. doi: 10.1186/s12874-019-0826-5 (PMC6751616; doi:10.1186/s12874-019-0826-5)
Supplement: Supplementary file 7 — Supplemental information’s on methods. Additional information on methods : (i) Method for comparison of the 3 different estimation ways used to fit the mixtures of Beta distributions; (ii) Definition of the non-informative and informative priors compared in the sensitivity analysis; (iii) Definition of the “good decision” for each scenario of the simulation study. (PDF 246 kb) [file 12874_2019_826_MOESM7_ESM.pdf]

Additional file 7.

Supplemental information on methods

1 Method for comparison of the 3 different estimation ways used to fit the mixtures of Beta distributions

To fit the acceptable difference between arms D_j as mixtures of beta regression using maximum likelihood estimation, we used the `betamix` function (`betareg` package on `R` software). The fitted distributions obtained through the `betamix` function, with 3 as the maximal number of components of the finite mixture, contained only 1 component for all but one event. To try to model those left long-tailed distributions (see for example Figure 2) by mixtures of 2 or 3 beta distributions, 3 different estimation methods of fitting (the first mathematically driven and the other two empirically driven) were applied (See Box 1 in the main manuscript) to model the distribution of physicians' acceptable differences of rate of events. The distributions obtained through the three methods were compared using criteria for goodness of fit based on area under curves.

The histogram of the acceptable differences among the E experts have been partitioned in $B = 101$ subintervals (known as bins) [-0.005, .005], [.005, .015], [.015, .025],. . ., [.995, 1.005]. For simplicity, even if the acceptable differences are distributed on (0, 1), the bins were constructed from -0.005 to 1.005 in order to be centred on the observed values $0, 0.01, 0.02$....., so that the histograms are not influenced by the right (or left)-closing of the intervals.

For each bin, the absolute area of the gap between the fit distribution and the histogram is calculated. Then, those absolute areas are summed. Denoting x_b the lower value of the bin and $y_b = x_b + 0,01$ the upper value of the bin, $h_b(x)$, the function that counts the number of observations that fall into the b bin, and $f(x)$, the model for the physicians' acceptable differences of rate of events fitted by one of the 3 methods described above $(f(a_{1,j}, b_{1,j}, a_{2,j}, b_{2,j}, a_{3,j}, b_{3,j}, w_{1,j}, w_{2,j}, w_{3,j})$, the criteria for goodness of fit of the fitted distribution $f(x)$ is then given by:

$$G(f) = \sum_{b=1}^{100} | \int_{x=x_b}^{y_b} f(x)\,\mathrm{dx} - \frac{h_b(x)}{\sum_{b=1}^{100} h_b(x)} |$$

Finally, for each event (j), the method that gave the function f(x) with the lowest $G(f)$ was retained as the distribution for D_j.

2 Definition of the priors

Thirteen pairs of priors $(\theta_{0,j}, \theta_{1,j})$ have been tested in the simulation study:

- A non-informative prior density, with $\alpha_{1,j} = \alpha_{0,j} = \beta_{1,j} = \beta_{0,j} = 1$
- Twelve informative priors:
  - In the full dose arm, we applied Beta$(\alpha_{0,j}, \beta_{0,j})$ coming from historical data [ref], with 4 different precisions: $\alpha_{0,j}$ and $\beta_{0,j}$ were respectively equal to the number of successes/failures in the EPIPAGE study, divided by 1, 3, 10, or 20.
  - In the half dose arm, we applied, for each of those precision's, 3 different means for the difference between the two arms: (i) no difference: $E(\pi_{1,j} - \pi_{0,j}) = 0$, (ii) a difference of failure equal to the median acceptable difference according to experts: $E(\pi_{1,j} - \pi_{0,j}) = \text{median}(d_{j,e})$, (iii) the number of failures in the half-dose arm was assumed to be twice than that in the full-dose arm: $E(\pi_{1,j} - \pi_{0,j}) = \pi_{0,j}$.

  Figure 1 gives a graphical representation of the 12 pairs of informative priors for severe intraventricular haemorrhage. The parameters of the 12 informative priors for the 4 events are summarized in Table 1.

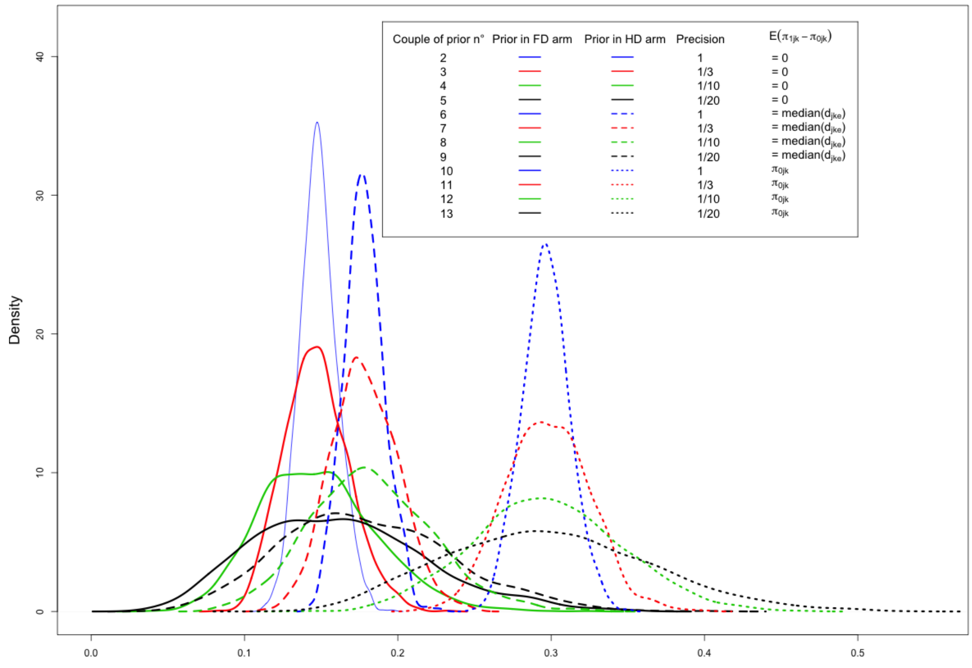

Figure 1: Plots of the prior distribution in the full-dose (FD) and in the half-dose (HD)arms for the 12 pairs of informative priors for severe intraventricular haemorrhage.

HD arm: half dose arm; FD arm: full dose arm

$\pi_{i,j}$: rate of event j in the ith arm

$d_{j,e}$: acceptable difference of event j between arms, according to the eth expert

Table 1: Parameters for the prior distribution in the full-dose (FD) and in the half-dose (HD) arms for the 13 pairs of priors according to the event.

**Prior**				**Parameters**							
				**Death**		**IVH**[1]		**NEC** [2]		**Retinopathy**	
N	Type	$E(\pi_{1,j} - \pi_{0,j})$ [1]	Precision	FD arm (a,b)	HD arm (a,b)	FD arm (a,b)	HD arm (a,b)	FD arm (a,b)	HD arm (a,b)	FD arm (a,b)	HD arm (a,b)
1	Non-informative			(1, 1)	(1, 1)	(1, 1)	(1, 1)	(1, 1)	(1, 1)	(1, 1)	(1, 1)
2	Informative	= 0	1	(573, 881)	(573, 881)	(130, 745)	(130, 745)	(45, 827)	(45, 827)	(37, 835)	(37, 835)
3	Informative		1/3	(191, 294)	(191, 294)	(43, 249)	(43, 249)	(15, 276)	(15, 276)	(12, 279)	(12, 279)
4	Informative		1/10	(57, 88)	(57, 88)	(13, 75)	(13, 75)	(4, 83)	(4, 83)	(4, 83)	(4, 83)
5	Informative		1/20	(29, 44)	(29, 44)	(7, 37)	(7, 37)	(2, 42)	(2, 42)	(2, 42)	(2, 42)
6	Informative	= median$(d_{j,e})$ [3]	1	(573, 881)	(631, 823)	(130, 745)	(156, 719)	(45, 827)	(62, 810)	(37, 835)	(54, 818)
7	Informative		1/3	(191, 294)	(211, 274)	(43, 249)	(52, 240)	(15, 276)	(21, 270)	(12, 279)	(18, 273)
8	Informative		1/10	(57, 88)	(63, 82)	(13, 75)	(16, 72)	(4, 83)	(6, 81)	(4, 83)	(5, 82)
9	Informative		1/20	(29, 44)	(32, 41)	(7, 37)	(8, 36)	(2, 42)	(3, 41)	(2, 42)	(3, 41)
10	Informative	$= \pi_{0,j}$ [4]	1	(573, 881)	(1146, 308)	(130, 745)	(260, 615)	(45, 827)	(90, 782)	(37, 835)	(74, 798)
11	Informative		1/3	(191, 294)	(382, 103)	(43, 249)	(87, 205)	(15, 276)	(30, 261)	(12, 279)	(25, 266)
12	Informative		1/10	(57, 88)	(114, 31)	(13, 75)	(26, 62)	(4, 83)	(9, 78)	(4, 83)	(7, 80)
13	Informative		1/20	(29, 44)	(58, 15)	(7, 37)	(13, 31)	(2, 42)	(5, 39)	(2, 42)	(4, 40)

HD arm: half-dose arm; FD arm: full dose arm
[1]IVH: Intraventricular hemorrhage
[2]NEC: Necrotizing enterocolitis
[3]$d_{j,e}$: Acceptable difference of event j between arms, according to the eth expert
[4]$\pi_{i,j}$: Rate of event j in the ith arm

3 Definition of the "good decision" for each scenario of the simulation study

To build the decision rule, we had to decide what was the "good decision" for each scenario, i.e., which scenario was unacceptable or not, according to the event. For each of the 5 scenarios, a 'standard' population of 3146 children, with an observed distribution of gestational ages and prevalence exactly equal to the expected (See Table 1 in the main manuscript), was built. For each scenario, we considered that the good decision was :

- to conclude that the difference was acceptable if the lower bound of the frequentist 95th confidence interval ($95CI$) for the difference in that standard population was lower or equal to the median acceptable difference among the experts,
- to conclude that the difference was unacceptable if it was higher.
