# Supplementary material for: A Bayesian non-inferiority approach using experts’ margin elicitation – application to the monitoring of safety events
Source: BMC Med Res Methodol. 2019 Sep 18;19:187. doi: 10.1186/s12874-019-0826-5 (PMC6751616; doi:10.1186/s12874-019-0826-5)
Supplement: Supplementary file 8 — Physician experts who participated in the elicitation, BETADOSE trial. Complete list of experts who participated in the elicitation. (PDF 41 kb) [file 12874_2019_826_MOESM8_ESM.pdf]

Physician experts who participated in the elicitation, BETADOSE trial.

Investigator	Hospital	City
ALEXANDRE Cénéric	CHU Caen Normandie	Caen
BALITALIKE Jadot	CH Valenciennes	Valenciennes
BAUD Olivier	HU Robert-Debré (APHP)	Paris
BLANC Julie	Hôpital Nord (AP-HM)	Marseille
BOIZE Philippe	CH René Dubos	Pontoise
BOHEC Caroline	CH Pau	Pau
BOUET Pierre-Emmanuel	CHU Angers	Angers
BOURTEMBOURG MATRAS Aude	CHRU Jean Minjoz	Besançon
BREVAUT-MALATY Véronique	Hôpital Nord (AP-HM)	Marseille
CAMBONIE Gilles	Hôpital Arnaud de Villeneuve - CHU Montpellier	Montpellier
CASPER Charlotte	Hôpital des enfants - CHU de Toulouse	Toulouse
CHAULEUR Céline	Hôpital Nord - CHU Saint Etienne	Saint Etienne
COSTE-MAZEAU Perrine	Hôpital Mère-Enfant - CHU de Limoges	Limoges
CUDEVILLE Corinne	CH Bretagne-Atlantique	Vannes
DE LUCA Daniele	Hôpital Antoine-Beclere - HU Paris Sud (APHP)	Clamart
DEBILLON Thierry	Hôpital Couple-Enfant - CHU Grenoble Alpes	Grenoble
DERUELLE Philippe	Hôpital Jeanne de Flandres - CRHU de Lille	Lille
DESFRERE Luc	Hôpital Louis-Mourier - HU Paris Nord Val-de-Seine (APHP)	Colombes
DI MAIO Massimo	HU Carémeau	Nîmes
DORET Muriel	Hôpital Femme-Mère-Enfant - Hospices Civils de Lyon	Bron
DREYFUS Michel	CHU Caen Normandie	Caen
DURRMEYER Xavier	Centre Hospitalier Intercommunal	Créteil
ECKMAN LACROIX Astrid	CHRU Jean Minjoz	Besançon
FLAMANT Cyril	Hôpital Mère-Enfant - CHU de Nantes	Nantes
GALLOT Denis	CHU Clermont-Ferrand	Clermont-Ferrand
GASCOIN Géraldine	CHU Angers	Angers
GOFFINET François	Hôpital Port-Royal - HU Paris Centre (APHP)	Paris
HECKENROTH Hélene	Hôpital de la Conception (AP-HM)	Marseille
HOULLIER Marie	Hôpital Bicêtre - HU Paris Sud (APHP)	Le Kremlin Bicêtre
JARREAU Pierre-Henri	Hôpital Port-Royal - HU Paris Centre (APHP)	Paris
LANGER Bruno	Hôpital de Hautepierre - HU Strasbourg	Strasbourg
LAPILLONNE Alexandre	Hôpital Necker - HU Paris Centre (APHP)	Paris
LE SACHE Nolwenn	Hôpital Bicêtre - HU Paris Sud (APHP)	Le Kremlin Bicêtre
MADELENEAU Doriane	CH Bretagne-Atlantique	Vannes
MANDELBROT Laurent	Hôpital Louis-Mourier - HU Paris Nord Val-de-Seine (APHP)	Colombes
PAGES Anne-Sophie	CH du Cotentin	Cherbourg
PATURAL Hugues	Hôpital Nord - CHU Saint Etienne	Saint Etienne
PONCELET Christophe	CH René Dubos	Pontoise
RAYSSIGUIER Romy	Hôpital Arnaud de Villeneuve	Montpellier
RIGONNOT Luc	Centre Hospitalier Sud Francilien	Corbeil-Essonnes
SCHMITZ Thomas	HU Robert-Debré (APHP)	Paris
SENTILHES Loic	CHU de Bordeaux	Bordeaux
TILLOUCHE Nadia	CH Valenciennes	Valenciennes
TOURNEUX Pierre	CHU Amiens-Picardie	Amiens
WASZAK Paul	Hôpital Delafontaine	Saint Denis
