# Supplementary material for: A Bayesian non-inferiority approach using experts’ margin elicitation – application to the monitoring of safety events
Source: BMC Med Res Methodol. 2019 Sep 18;19:187. doi: 10.1186/s12874-019-0826-5 (PMC6751616; doi:10.1186/s12874-019-0826-5)
Supplement: Supplementary file 9 — Comparison of the use of a mixture distribution versus the use of an average value of elicitated margins from experts. In this additional work, two approaches were proposed to compute the acceptable difference from experts’ elicitation: the method described in the main manuscript and the use of average values. (PDF 368 kb) [file 12874_2019_826_MOESM9_ESM.pdf]

Additional file 9.

Comparison of mixture distributions versus average value of elicitated margins from experts.

1 Methods

In this additional work, two approaches were proposed to compute the acceptable difference from experts' elicitation: the method described in the main manuscript and the use of average values. To simplify the notations, we will give the theoretical basis on a single event. Let d_e denote the acceptable difference between arms, according to the eth expert, $e \in [1, \ldots, E]$.

- If the acceptable difference D is fitted through a mixture distribution, as described in the main manuscript, the posterior probability that the difference of event rates, $\theta_1 - \theta_0$, is higher than the acceptable difference at the final analysis (l=11) is as follows:

$$\begin{aligned} P(\delta^{11}) &= P(\theta_1 - \theta_0 > D \mid Y_1^{11}, Y_0^{11}) \\ &= \int_0^1 (\theta_1 - \theta_0 > x \mid Y_1^{11}, Y_0^{11}, D = x) \, . \, P(D = x) \ \mathrm{dx} \end{aligned} \tag{1}$$

  with $D \sim f(a_1, b_1, a_2, b_2, a_3, b_3, w_1, w_2, w_3)$ computed from experts elicitation using the `betareg` package on `R` software (see more details in main manuscript).

- In the second case, if the acceptable difference is estimated using the average of all experts' opinion, the posterior probability that the difference of event rates, $\theta_1 - \theta_0$, is higher than the acceptable difference is as follows:

$$\begin{aligned} P(\delta^{11}) &= P(\theta_1 - \theta_0 > D \mid Y_1^{11}, Y_0^{11}) \\ &= P(\theta_1 - \theta_0 > \frac{\sum d_e}{E} \mid Y_1^{11}, Y_0^{11}) \end{aligned} \tag{2}$$

Simulation study

In an extensive simulation study, we compared the results obtained by these two approaches. Taking inspiration of our study, we performed simulations using the prevalence of neonatal death ($\theta_0 = 0.39$) in trials with same sample size ($n_i = 162$). To simulate the data of the trials, 5 scenarios have been used. Let T1, T2, T3, T4 and T5 denote the different scenarios. In all scenarios, the prevalence is higher in the experimental than in the control arm ($\theta_{1,T1} = 1.2 \times \theta_0$, $\theta_{1,T2} = 1.5 \times \theta_0$, $\theta_{1,T3} = 1.6 \times \theta_0$, $\theta_{1,T4} = 1.7 \times \theta_0$, and $\theta_{1,T5} = 2.0 \times \theta_0$). For each scenario, 1000 trials have been generated.

To address the issues of how acceptable difference D and posterior probabilities are affected by the two approaches, we constructed an array of 3 data sets of experts'

elicitation, with $E = 44$, each obtained by specifying 1 or 2 *Beta* functions. Let E1, E2 and E3 denote the 3 data sets of experts' elicitation. The parameters have been chosen in order to have an average equal to 0.20 in the 3 data sets:

- In the first data set of experts (E1), $d_e \sim Beta(10, 40)$, with expected value $\mu = 0.20$ and precision parameter $\Phi = 50$. In this data set, the experts seem to agree about the margin and the distribution is uni-modal with small variability.
- In the second data set of experts (E2), $d_e \sim Beta(1, 4)$, with expected value $\mu = 0.20$ and precision parameter $\Phi = 5$. In this data set, the experts disagree about the margin and the variability is high.
- In the third data set of experts (E3), half of the answers were distributed through $d_e \sim Beta(0.5, 49.5)$, with expected value $\mu = 0.01$ and precision parameter $\Phi = 50$ and half through $d_e \sim Beta(19.5, 30.5)$ with expected value $\mu = 0.39$ and precision parameter $\Phi = 50$. In this data set, the experts disagree about the margin and the distribution seems to be bi-modal.

The figure 1 presents the histogram of the acceptable difference of death among the E experts (d_e), in the 3 proposed data sets.

The posterior probability that observed differences is higher than the acceptable difference was calculated following the equation (1) and (2), for each method, each data set of experts (E1, E2, E3) and each trial of each scenario (T1, T2, T3, T4, T5). Then we calculated the overall number of conclusions obtained when applying decision thresholds of 0.50 at the final analysis.

2 Results

The figure 1 presents, for each of the 3 data sets of experts, the histogram of the acceptable difference of death among the E experts (d_e), the mean of the difference, and the fit (D) obtained using our proposed method.

The Table 1 summarizes the posterior probabilities and the conclusions of the decision rule according to the 2 approaches, for each data set of experts and each scenario. The Figure 2 compares the conclusions of the trials given by the 2 approaches, using a decision threshold of 0.50, for each data set of experts and each scenario. The results regarding the decision rule for the average are very close across data sets of experts, which is not the case for the mixture. The Figure 3 compares the posterior probabilities given the 2 approaches, for each data set of experts and each scenario. In this figure the reader can have a detailed view on how the average and the mixture influence the distribution of the posterior probability.

For all the scenarios, if we compute the acceptable difference as an average, the decision that the difference was unacceptable occurs among the same proportion of trials, using the 3 data sets of experts, even though the consensus between experts was not the same between these 3 situations. The distribution and heterogeneity of experts' opinion is not taken into account in this case. Conversely, if we compute the acceptable difference as a mixture, the decision was varying according to the data set of experts. The higher difference between the two approaches was observed

under the second data set of experts, and the lower under the first data set of experts in which the variability was small. Finally, using 3 data sets with almost same mean acceptable difference of experts (0.20), the 2 methods gave different results, and the difference between the two methods increased as the variability among experts increased.

3 Interpretation

The use of mixture of *Beta* distributions seemed better to capture experts' opinions, at least in situations where the variability across experts' opinion was high. When data aren't available for fixing the margin before the trial onset, an elicitation process among experts can help to reflect the belief about the experimental treatment and the margin. The main concern is about how uncertain the investigators are before fixing the trial design and how this uncertainty can be reflected in the final trial design. The uncertainty analysis and its quantification are important issues that need to be addressed. In the field of clinical trial, investigators are often far from the concept of "equipoise" but, on the opposite, they are still unsure about some design's choices. The Bayesian inference allows to take this into account and to reflect this in the data analysis. Our approach is one proposition among several possibles, we have shown that experts do not necessary always agree and that using an average value or using a Delphi process, in order to get one consensus, could not be the best way to follow when designing clinical trial.

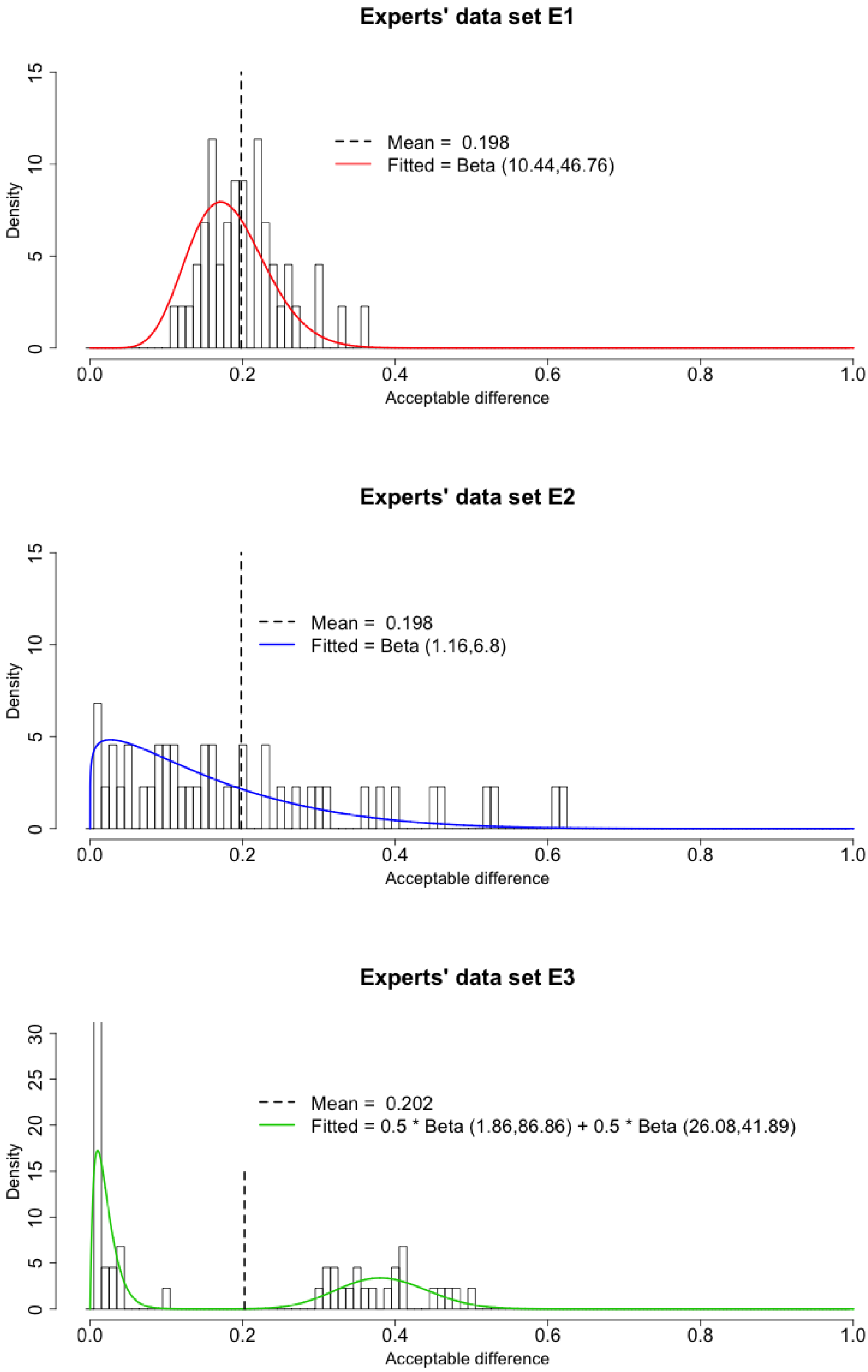

Figure 1: **Histogram of the acceptable difference between arms, and mixtures of Beta distributions fitted from experts' elicitation, for the three data sets of experts. All three histograms share the same mean, that is, 0.2.**

The histograms represent the acceptable difference among the E experts (d_e). The lines represent the fits of this difference (D), obtained through mixtures of Beta distributions. The legend presents the average of the difference among the E experts and the parameters of the fits.

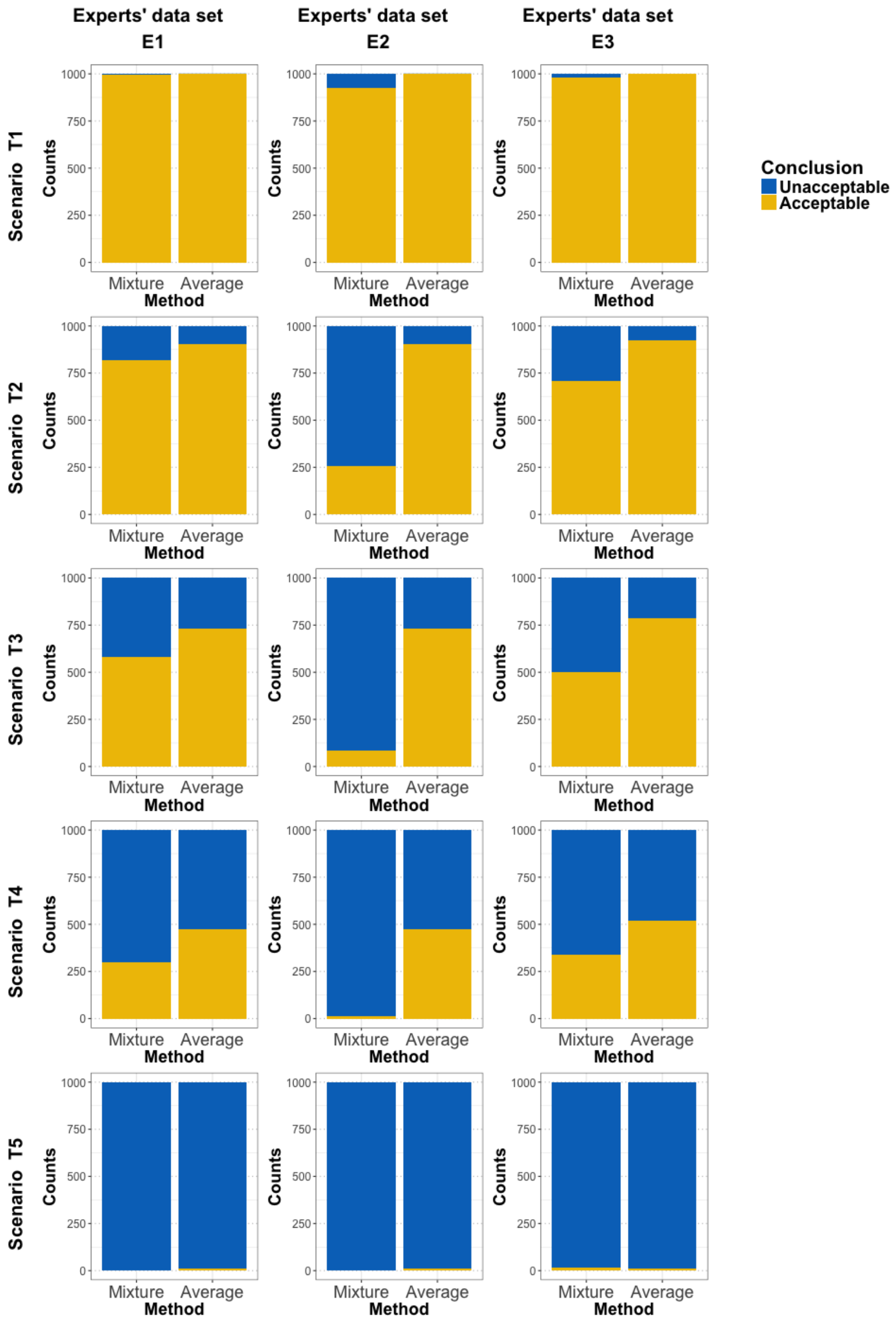

Figure 2: **Barplot of the conclusions of the trials obtained with acceptable difference computed as a mixture distribution or as an average value, using a decision threshold of 0.50, according to the experts' data set and to the scenario.**

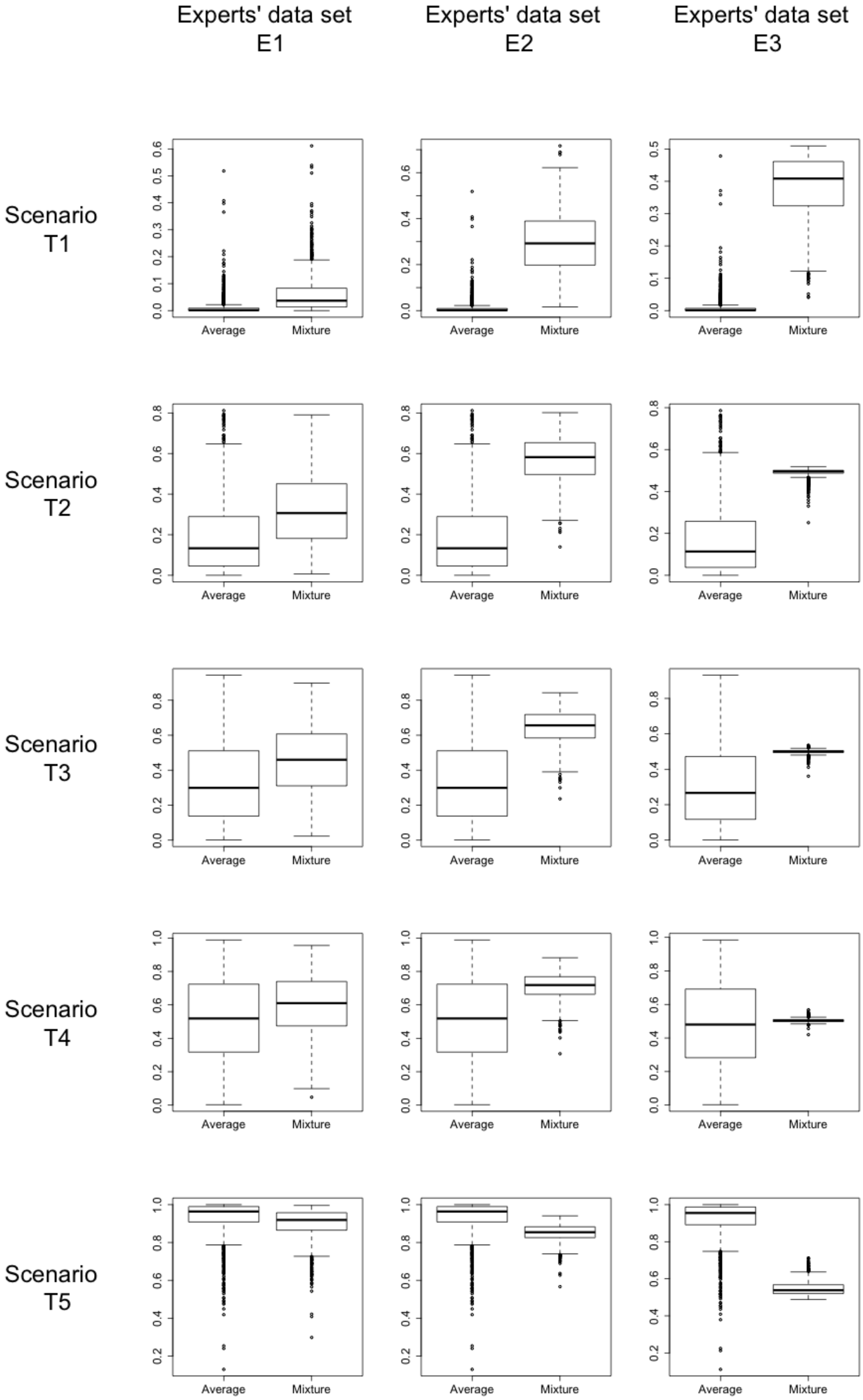

Figure 3: **Boxplot of the posterior probabilities obtained with acceptable difference computed as a mixture distribution or as an average value, according to the experts' data set and to the scenario.**

Table 1: **Posterior probability and decision obtained using acceptable difference computed as a mixture distribution or an average value, according to the experts' data set and to the scenario.**

**Data set for experts'** [1]	**Scenario** [2]	**Acceptable difference as distribution**		**Acceptable difference as average**	
		Posterior probability Median (IQR)	Trials with decision = Unacceptable [3] N = 1000	Posterior probability Median (IQR).	Trials with decision = Unacceptable [3] N = 1000
E1	T1	0.04 [0.01 ; 0.08]	4	0.00 [0.00 ; 0.01]	1
	T2	0.31 [0.18 ; 0.45]	182	0.13 [0.05 ; 0.29]	95
	T3	0.46 [0.31 ; 0.61]	420	0.30 [0.14 ; 0.51]	266
	T4	0.61 [0.47 ; 0.74]	700	0.52 [0.32 ; 0.72]	524
	T5	0.92 [0.87 ; 0.96]	997	0.96 [0.91 ; 0.99]	991
E2	T1	0.29 [0.20 ; 0.39]	75	0.00 [0.00 ; 0.01]	1
	T2	0.58 [0.50 ; 0.65]	742	0.13 [0.05 ; 0.29]	95
	T3	0.66 [0.58 ; 0.72]	915	0.30 [0.14 ; 0.51]	266
	T4	0.72 [0.66 ; 0.77]	989	0.52 [0.32 ; 0.72]	524
	T5	0.85 [0.83 ; 0.88]	1000	0.96 [0.91 ; 0.99]	991
E3	T1	0.41 [0.32 ; 0.46]	18	0.00 [0.00 ; 0.01]	0
	T2	0.50 [0.49 ; 0.50]	294	0.11 [0.04 ; 0.26]	76
	T3	0.50 [0.49 ; 0.50]	499	0.27 [0.12 ; 0.47]	213
	T4	0.50 [0.50 ; 0.51]	661	0.48 [0.28 ; 0.69]	482
	T5	0.54 [0.52 ; 0.57]	985	0.96 [0.89 ; 0.99]	988

IQR: Interquartile range

[1]E1: $d_e \sim Beta(10, 40)$; E2: $d_e \sim Beta(1, 4)$; E3: $d_e \sim 0.5 * Beta(0.5, 49.5) + 0.5 * Beta(19.5, 30.5)$

[2]T1: $\theta_{1,T1} = 1.2 \times \theta_0$; T2: $\theta_{1,T2} = 1.5 \times \theta_0$; T3: $\theta_{1,T3} = 1.6 \times \theta_0$; T4: $\theta_{1,T4} = 1.7 \times \theta_0$; T5: $\theta_{1,T5} = 12.0 \times \theta_0$; ; For each scenario, 1000 trials have been generated, with $n_i = 162$

[3]The decision rule conclude that the difference is higher than the acceptable difference if posterior ≥ 0.50
